# Tobacco package health warnings about product manipulations: an experimental study among Australian adults who smoke

**DOI:** 10.1093/heapro/daae210

**Published:** 2025-04-03

**Authors:** Emily Brennan, Claudia Gascoyne, Kimberley Dunstone, Amanda Vittiglia, Gayathri Srinivasan, James F Thrasher, Janet Hoek, Melanie Wakefield, Sarah Durkin

**Affiliations:** Centre for Behavioural Research in Cancer, Cancer Council Victoria, Level 8, 200 Victoria Parade, Naarm, East Melbourne, Victoria, 3002, Australia; Melbourne School of Psychological Sciences, University of Melbourne, Grattan St, Naarm, Parkville, Victoria, 3010, Australia; Centre for Behavioural Research in Cancer, Cancer Council Victoria, Level 8, 200 Victoria Parade, Naarm, East Melbourne, Victoria, 3002, Australia; Institute for Global Health, University College London, 90 Tottenham Court Road, London, W1T 4TJ, United Kingdom; Centre for Behavioural Research in Cancer, Cancer Council Victoria, Level 8, 200 Victoria Parade, Naarm, East Melbourne, Victoria, 3002, Australia; Australian Institute of Family Studies, Level 4, 40 City Road, Naarm, Southbank, Victoria, 3006, Australia; City of Port Phillip, 99a Carlisle Street, Naarm, St Kilda, Victoria, 3182, Australia; Department of Health Promotion, Education and Behaviour, Arnold School of Public Health, University of South Carolina, 921 Assembly Street, Columbia, SC, 29208, United States; Department of Public Health, University of Otago, Level 4, 29 Brandon Street, Wellington Central, Wellington 6011, Aotearoa, New Zealand; Centre for Behavioural Research in Cancer, Cancer Council Victoria, Level 8, 200 Victoria Parade, Naarm, East Melbourne, Victoria, 3002, Australia; Melbourne School of Psychological Sciences, University of Melbourne, Grattan St, Naarm, Parkville, Victoria, 3010, Australia; Centre for Behavioural Research in Cancer, Cancer Council Victoria, Level 8, 200 Victoria Parade, Naarm, East Melbourne, Victoria, 3002, Australia; Melbourne School of Psychological Sciences, University of Melbourne, Grattan St, Naarm, Parkville, Victoria, 3010, Australia

**Keywords:** tobacco, warnings, packaging, misperception, experimental study, additives/constituents, filter ventilation, roll-your-own tobacco, menthol, combustion

## Abstract

The tobacco industry manipulates cigarettes to provide sensory cues that mislead people who smoke. We assessed the effectiveness of health warnings (HWs) focussed on the impact of misleading product attributes, including filter ventilation, menthol, and roll-your-own tobacco, which were called Product Attribute HWs. Australian adults who smoke (*n* = 2544) were randomly assigned to view: control medication warnings; new Standard HWs with smoking harm images; Product Attribute HWs; or Product Attribute HWs plus a video. At baseline, participants were exposed to seven condition-specific warnings (+ video for those in the Product Attribute HWs + Video condition), then they were potentially re-exposed to one warning daily for 7 days (+ up to six video viewings). Participants (*n* = 1414) were followed-up at 8 days. Compared to controls, exposure to Product Attribute HWs elicited: greater increases in concern when inhaling smoke with misleading favourable (e.g. smooth) and unfavourable (e.g. harsh) sensory cues; greater knowledge of industry manipulation; greater self-centric and industry-centric negative emotions; higher product-specific smoking dissonance; and a higher likelihood of past-week rumination about HWs. Compared to Standard HWs, Product Attribute HWs elicited greater knowledge of product manipulation and industry-centric negative emotions, but similar self-centric negative emotions and past-week rumination about warnings. Only the Product Attribute HWs + Video condition resulted in greater discussion and online information-seeking about warnings. Product Attribute HWs that challenge long-standing myths fostered by tobacco companies yield many similar outcomes to Standard HWs but also provide unique beneficial outcomes. Nations should consider including Product Attribute HWs in their suites of tobacco HWs.

**Contribution to Health Promotion**

- This study tested the effects of a set of novel pack warnings that explain how the tobacco industry manipulates cigarettes and tobacco to provide pleasant sensations (e.g. the smooth or fresh taste of the smoke) that mislead people into believing smoking is less harmful.
- Product manipulation warnings performed in many similar positive ways to standard tobacco health harm warnings.
- Product manipulation warnings also increased knowledge about and negative emotions towards the tobacco industry, which a related study found predicts subsequent quitting behaviours.
- Nations should consider including product manipulation warnings in their sets of tobacco health warnings.

## BACKGROUND

The tobacco industry has long manipulated specific tobacco product attributes to invite incorrect inferences of lower harm (National Cancer Institute 2001). For example, introduced from the late 1960s, filter-ventilation—tiny vents around the filter tipping paper that dilute the inhaled smoke with air—makes cigarettes taste smoother or lighter (Kozlowski and O’Connor 2002). A flawed industry-promoted laboratory method suggested that filter ventilation lowers the yield of tar, nicotine, and carbon monoxide (National Cancer Institute 2001). However, most people who smoke partially block the vents with their fingers or mouths, smoke more intensively, or otherwise increase the smoke volume inhaled in pursuit of their desired nicotine delivery (National Cancer Institute 2001, Kozlowski and O’Connor 2002). These compensatory practices explain why filter ventilation does not reduce human toxicant exposure or harm, while creating sensory cues of smoke smoothness or lightness that generate a misperception of reduced harm (Carroll et al., 2021). The sensation of smoothness or lightness produced by filter-ventilation drives reduced harm beliefs (Elton-Marshall et al. 2015, King et al. 2023a). These lighter-tasting cigarettes were originally marketed as ‘low tar’, ‘mild’, or ‘light’ cigarettes (National Cancer Institute 2001, King et al. 2003; Cataldo and Malone 2008). In nations where these misleading terms were banned, tobacco companies used ‘smooth’ descriptors or lighter-coloured packs in white, silver, or gold to connote reduced harshness and lower harm (Mutti et al. 2011, Yong et al. 2016, Lempert and Glantz 2017). Where tobacco plain packaging has standardized packs to a single colour, tobacco companies have used lighter-colour brand variant names (Moodie et al. 2019).

Tobacco companies began using menthol as a fresh-tasting additive in the 1950s to allay health fears (Anderson 2011). Menthol cigarettes are associated with inferences of reduced harm, most likely due to their ‘fresh’ taste and smooth, throat-soothing sensation (Wilson et al. 2011, Brennan et al. 2015, Wackowski et al. 2018, Mancuso et al. 2021), even though they are no less harmful than non-menthol cigarettes and may make tobacco more addictive and smoking harder to quit (Villanti et al. 2017, Smith et al. 2020, Wickham 2020). Likewise, roll-your-own (RYO) tobacco is incorrectly perceived as more natural and less harmful than tailor-made cigarettes (Young et al. 2010, Filippidis et al. 2020, Moodie and O’Donnell 2022), a perception driven by the moistness of RYO tobacco and the mistaken inference that it has fewer additives and is less processed than tailor-made cigarettes (Edwards 2014, Hoek et al. 2017, Breslin et al. 2018). People who are reassured by misleading features and switch to or continue smoking these products may see quitting as less urgent or important (Kozlowski et al. 1998, Tindle et al. 2006).

Options for regulating product attractiveness, including the above product manipulations, have been explored by several expert advisory groups (Scientific Committee on Emerging and Newly Identified Health Risks (SCENIHR) 2016, World Health Organization Study Group on Tobacco Product Regulation (TobReg) 2018, 2019). To date, Articles 9 and 10 of the Framework Convention on Tobacco Control comprise only partial guidelines for regulating product design and attractiveness and limiting industry disclosure about product design and attractiveness (World Health Organization Framework Convention on Tobacco Control 2017). Only a few nations have banned menthol in tobacco products, and no nation has yet banned or comprehensively addressed other misleading product attributes such as filter-venting or RYO tobacco. In this context, education may help build momentum for policy change. To date, most public education efforts (Kozlowski et al. 2000, Australian Competition & Consumer Commission 2005, Edwards 2014, Kostygina et al. 2020, New York State Department of Health 2023) and research studies (Kozlowski et al. 1999, Kozlowski et al. 2001, Shiffman et al. 2001, Lee et al. 2019) have used media campaigns or corrective messages to address single product attribute misperceptions. However, including corrective information about the misleading nature of product attributes within sets of pictorial health warning (HW) rotations could address multiple misperceptions and potentially offer frequent message exposure near the time of purchase and use. Pictorial HWs about specific tobacco harms have been implemented in many countries (Canadian Cancer Society 2023) and have been shown to increase awareness of smoking harms, help-seeking to quit, early cognitive, affective, and behavioural predictors of later quit attempts, and smoking cessation (Thrasher et al. 2019). These Standard HWs also have beneficial effects on many vulnerable population subgroups, including people who are disadvantaged (Thrasher et al. 2012). To the best of our knowledge, researchers have not yet systematically assessed the effectiveness of HWs focussed on misleading product attributes and associated sensory experiences.

In this study, we developed a series of new HWs, which were called Product Attribute HWs, given their focus on the ways in which tobacco products are manipulated to create misleading sensory experiences. The development of these Product Attribute HWs was informed by recent developments in the field of misconception correction (Lewandowsky et al. 2012, Cappella et al. 2015, Cook and Lewandowsky 2020). This work suggests correcting misperceptions requires an understanding of the underlying rationale (e.g. ‘It feels smooth so it can’t be that harmful’) and then providing a plausible and evidenced alternative explanation (e.g. ‘Smooth feeling smoke is just as harmful, because the smooth feeling is created by mixing additives into the tobacco to mask the harshness’). Accordingly, our Product Attribute HWs focussed on acknowledging the compelling sensory cues that distract from smoking’s harms (smooth or light smoke for filter-venting; fresh clean taste for menthol; moist and natural-looking RYO tobacco) and reframing their meaning. Separate Product Attribute HWs explained how tobacco manufacturers generate these cues by manipulating products to mask the underlying harshness of tobacco smoke, a sensory cue that would otherwise signal bodily danger. We also addressed an underlying misconception that much of tobacco-caused harm is attributable to additives, rather than combustion of the tobacco (King et al. 2019, King et al. 2023b).

Our experimental study aimed to determine the potential effectiveness of Product Attribute HWs. As the effectiveness of Standard HWs is well established (Thrasher et al. 2019), we assessed whether the Product Attribute HWs were at least as effective if not more impactful than the Standard HWs across a range of outcome measures. To do this, we first compared both the Product Attribute HWs and Standard HWs to a control condition to examine the effects of exposure to each type of HW. Second, we compared the effects of exposure to the Standard HWs to those of exposure to the Product Attribute HWs. Furthermore, we also aimed to determine whether a video advertisement complementing the Product Attribute HWs could augment the effects of exposure to Product Attribute HWs alone. Public education campaigns to complement Standard HWs amplify awareness of smoking harms (Thrasher et al. 2019), intentions to quit (Brennan et al. 2011), awareness of a toll-free quitline (Thrasher et al. 2013), and increase discussion about featured harms (Nagelhout et al. 2015), which predicts quit attempts (Thrasher et al. 2016). Video-based campaigns may provide critical complements for Product Attribute HWs if people who smoke need additional illustration or an affective elicitation to counter entrenched misperceptions (Cook and Lewandowsky 2020).

## METHODS

### Study design

**Figure 1.**
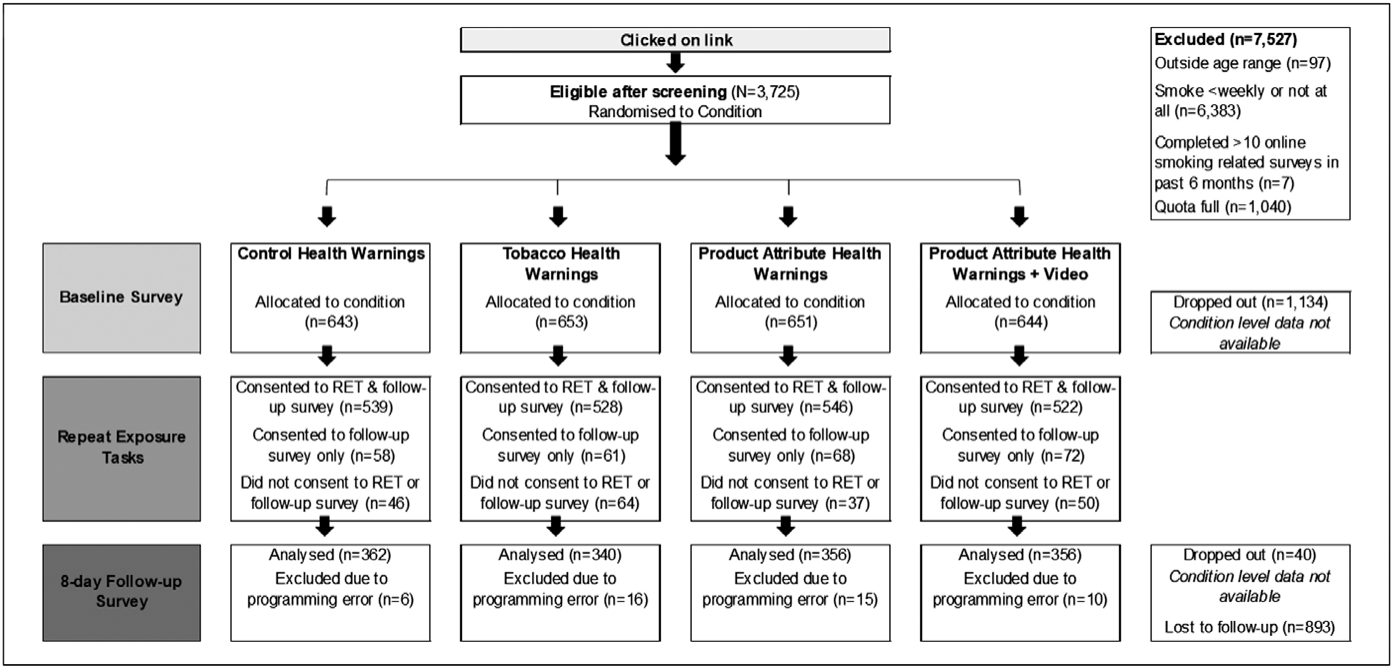
Participant flow diagram.

We conducted a four-arm between-subjects online experimental study. Eligible participants were stratified based on whether they predominantly smoked factory-made cigarettes (FMC), RYO cigarettes, or menthol cigarettes and then randomized using the least-filled quota method to either: (i) control warnings on over-the-counter medication packaging; (ii) Standard HWs with messages and imagery of smoking-related health harms; (iii) Product Attribute HWs with messages and imagery about tobacco product attributes that mislead or distract from harms; or (iv) Product Attribute HWs plus a brief video reinforcing the main messages from the warnings. Participants were exposed online to their assigned stimuli within a baseline survey, and then potentially repeatedly exposed to the stimuli online over the next 7 days through a series of repeated exposure tasks that participants could opt-in to complete. This approach aimed to simulate real-life repeated exposure to HWs and video advertisements. We conducted a follow-up survey at 8 days (see Fig. 1).

The study was approved by Cancer Council Victoria’s Human Research Ethics Committee. The study was prospectively registered in the Australian and New Zealand Clinical Trials Registry (ACTRN12622000581763).

### Participants

Data were collected between 3 August and 30 October 2022. Participants were recruited from Australian online non-probability panels sourced through an International Standards Organisation-accredited data collection agency. Eligible participants were 18 to 69 years old and currently smoked FMC and/or RYO cigarettes at least weekly. We aimed to achieve a proportionate stratified sample; soft quotas were applied for gender and age groups (non-intersecting, +/− 5% tolerance) within hard quotas for predominant product use (21% predominantly RYO, 54% predominantly FMC, 25% predominantly menthol) (Bayly et al. 2023).

Based on HW research led by one of our co-investigators (Thrasher et al. 2024) from which we calculated 1-week outcomes, we aimed to detect a 10%-point increase in forgoing a cigarette at follow-up between control vs. Product Attribute HWs. Power calculations indicated that *n* = 383 participants per condition would detect this difference at 90% power and α = 0.05 (two-tailed). We therefore aimed to recruit *N* = 2400 participants (*n* = 600 per condition) during the baseline survey, assuming a 64% retention rate at the 8-day follow-up. The planned sample size was also adequate to detect a 14%-point difference in performing any one of three smoke-limiting micro-behaviours at follow-up between the Product Attribute HWs + Video and Product Attribute HWs conditions (Unpublished data: Mitsopoulos, Vittiglia, & Durkin, 2021. *Outcome evaluation of the Sticky Blood campaign (2021)*. Centre for Behavioural Research in Cancer, Cancer Council Victoria. Melbourne, Australia).

### Interventions

**Figure 2.**
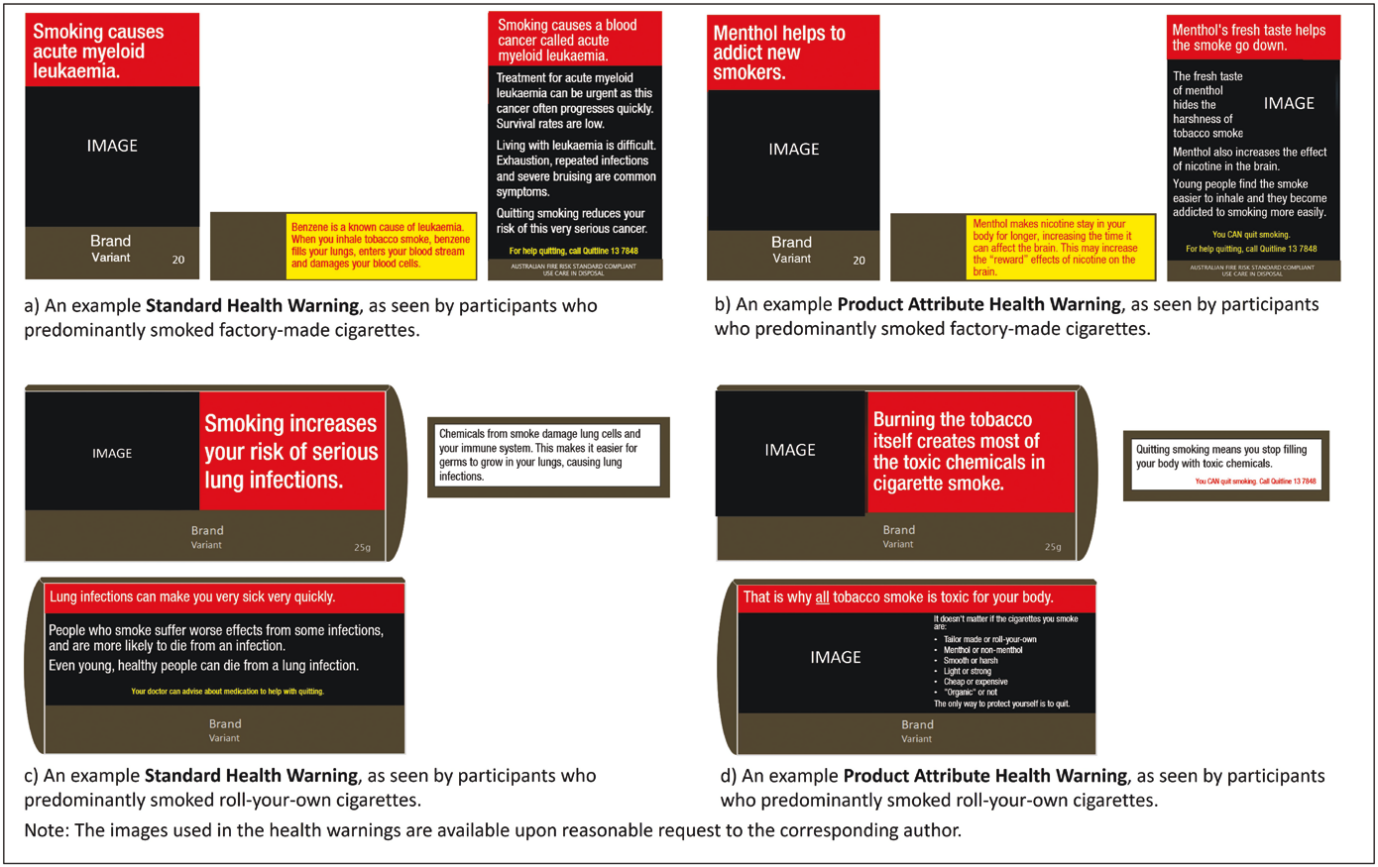
Examples of the Standard HWs and Product Attribute HWs (A copy of all Standard Health Warnings and all Product Attribute Health Warnings, including the images used, are available upon reasonable request to the corresponding author. A copy of the images used in the control condition is also available upon request. See Supplementary File Table S1 for the text featured in all 11 of the Product Attribute Health Warnings).

Control participants were exposed to images of popular non-prescription medications available in Australia (e.g. pain relief medication, anti-inflammatory treatments; images used are available upon reasonable request to the corresponding author). Participants in the Standard HWs condition were exposed to text and pictorial HWs that highlighted serious smoking-caused diseases using new content not previously featured in Australian HWs. For context, large graphic Standard HWs have been in force on plain packs in Australia since late 2012, addressing 14 smoking-related conditions in two annually rotating sets of seven HWs (Scollo et al. 2019). Research has demonstrated that the current HWs have been effective but have worn out over time (Essence Communications 2018, Swayampakala et al. 2018). The new Standard HWs used in the present study were developed in collaboration with a team with expertise in tobacco warning labels and science communication, and were designed to align with the specific smoking harms mentioned in the Product Attribute HWs as much as possible. Two examples of the Standard HWs (text only) are provided in Fig. 2 (a copy of all Standard HWs, including the images used, is available upon reasonable request to the corresponding author).

Participants in the Product Attribute HWs and Product Attribute HWs + Video conditions were exposed to text and pictorial HWs highlighting product attributes, sensory experiences, and other manipulations that may mislead people who smoke into believing certain products offer reduced harm. The Product Attribute HWs were developed through iterative research involving six sequential studies: the objectives, methods, and findings from each of these six studies are summarized in Supplementary Section 1. The content of the Product Attribute HWs evolved over the course of these studies, with some HWs removed from consideration and others added, eventually resulting in a final set of 11 Product Attribute HWs. Two of these HWs focussed on the use of filter-ventilation to yield smoother and lighter-feeling smoke. Three HWs focussed on the use of menthol to make smoke taste fresh, and an additional three HWs focussed on the use of humectants to moisten RYO tobacco and imply naturalness. The remaining three HWs aimed to establish the foundational understanding that most of the harm from tobacco smoke comes from combustion, not from additives, and to reinforce that additives are mostly included to mask the harshness of smoke so that it can be more easily inhaled. Two examples of the Product Attribute HWs (text only) are presented in Fig. 2 and the text included in all 11 Product Attribute HWs is provided in Supplementary Table S1 (a copy of all Product Attribute HWs, including the images used, is available upon reasonable request to the corresponding author). The final stage of the message development research was used to confirm that each of the Standard HWs and Product Attribute HWs developed for the study satisfied standard perceived effectiveness criteria (Noar et al. 2020) and improved relevant understanding (See Supplementary Section 1, Study 6).

All HWs were displayed on Australian plain standardized packs and generally featured an image with a headline statement or question on the front of pack, a different image with explanatory text on the back of pack, and additional explanatory text on the side of pack (or under the flap for RYO pouches). For all HWs, participants were progressively shown images of the front, back, and side (or under flap) of packaging, and all angles together, for a minimum of six seconds each. They were instructed to click to enlarge all images if needed.

Participants in the Product Attribute HWs and Product Attribute HWs + Video conditions were exposed to seven HWs in total: three Product Attribute HWs common to all, two Product Attribute HWs specifically relevant to participants’ predominant product use, and two Product Attribute HWs featuring non-predominant tobacco products to both simulate real-world exposure to a variety of HWs and convey that product switching would not reduce harms. They were exposed to each HW once during the baseline survey, and potentially re-exposed to each HW once again during the repeated exposure tasks. See Supplementary Table S2 for a description of which product HWs were shown to participants in the Product Attribute HWs and Product Attribute HWs + Video conditions based on the predominant tobacco product they smoked.

Participants in the Product Attribute HWs + Video condition were additionally exposed to a 30-second video advertisement developed for this study. The voiceover explained that tobacco companies purposefully use masking agents (additives that smooth the smoke), flavours, and modified filters to hide the harsh feel and taste of burning tobacco. Imagery featured rusted barbed wire concealed in cotton wool within a cigarette. As the cigarette was lit, the barbed wire was unwittingly inhaled. Participants were exposed twice to a 30-second video version during the baseline survey and potentially exposed to one of two randomly assigned 15-second versions once or twice during the repeated exposure tasks. See Supplementary Table S3 for all video descriptions and transcripts.

### Procedures

Online panel members were offered a reward value of up to AUD $18 to participate in the study. After clicking through and completing eligibility screening questions, participants provided consent.

Participants were randomized to condition, and then within each condition, participants were randomized to view 7 out of the possible 11 HWs relevant to that condition during baseline.

After baseline survey completion, participants were asked to consent to take part in daily repeated exposure tasks and/or the 8-day follow-up survey, and offered an additional reward value of up to AUD $16.50. Those who participated in the repeated exposure tasks were potentially exposed to their seven assigned HWs once during each daily survey. Those who consented received the follow-up survey invitation by email 8 days after completing the baseline survey, irrespective of whether they completed any repeated exposure tasks.

### Measures

#### Primary outcomes

We assessed *change in enjoyment* when inhaling smoke by asking participants at baseline prior to HW exposure and then again at follow-up to report how enjoyable they find inhaling smoke that had varied sensory cues. Participants used a sliding visual analogue scale anchored at 1 (‘not at all enjoyable’) and 100 (‘very enjoyable’). Favourable sensory cues comprised (i) smoke that feels light, (ii) smoke that feels smooth, (iii) smoke that has a fresh taste, and (iv) smoke that is soothing on the throat. These four items were averaged into one continuous measure (Cronbach’s α = 0.773). Unfavourable sensory cues comprised (i) smoke that feels heavy, (ii) smoke that feels harsh, (iii) smoke that has a tobacco taste, and (iv) smoke that is irritating on the throat, combined into one continuous measure (α = 0.763). *Change in concern* when inhaling smoke was measured similarly by asking participants pre-HW-exposure and then again at follow-up to rate the level of concern associated with the same favourable (i.e. light, smooth, fresh taste, soothing; α = 0.910) and unfavourable (i.e. heavy, harsh, tobacco taste, irritating; α = 0.746) sensory cues, anchored at 1 (‘not at all concerned’) and 100 (‘very concerned’).

Knowledge of harms despite sensory cues, knowledge of tobacco industry manipulation, self-centric negative emotional responses to HWs, industry-centric negative emotional responses to HWs, and product-specific smoking dissonance items, were each measured at follow-up using Likert scales (1 = ‘strongly disagree’; 5 = ‘strongly agree’) combined to create a mean score for each construct. Knowledge of harms despite sensory cues comprised three measures: (i) ‘no matter how it feels, all cigarette smoke is just as damaging’, (ii) ‘no matter how it tastes, all cigarette smoke is just as damaging’, and (iii) ‘no matter how the smoke feels or tastes, when I inhale cigarette smoke, I inhale hundreds of toxic chemicals’ (α = 0.867). Knowledge of tobacco industry manipulation comprised two measures: (i) ‘tobacco companies process raw tobacco to change the way the cigarette smoke feels and tastes’, and (ii) ‘tobacco companies modify cigarettes to change the way the cigarette smoke feels and tastes’ (α = 0.776). Self-centric negative emotional responses to HWs comprised three measures that the HWs (and video for the Product Attribute HWs + Video participants) made them feel (i) uncomfortable, (ii) worried, and (iii) embarrassed (α = 0.835). Industry-centric negative emotional response to HWs comprised two measures that the HWs (and video for the Product Attribute HWs + Video participants) made them feel (i) angry at tobacco companies and (ii) deceived by tobacco companies (α = 0.827). The product-specific smoking dissonance scale comprised five measures. Three items asked participants to consider how they had felt as they inhaled the smoke from their cigarettes over the past week: (i) found it less enjoyable than before, (ii) felt more uncomfortable about their smoking than before, and (iii) felt more uneasy about their cigarettes than before. Two items asked the extent to which participants had, in the past week, (iv) thought about the harm caused by their tobacco product more than they used to, and (v) felt put off from continuing to smoke their current tobacco product (α = 0.881).

Rumination about HWs was measured at follow-up by asking how often the HW messages or images came to mind in the past week, dichotomized into less than daily (comprised of ‘not at all’, ‘once over the week’, and ‘once every few days’) and at least once a day (comprising ‘once a day’ and ‘several times a day’). Online searching related to the HWs was measured by asking whether participants (‘yes/no’) had searched for information online about the messages and images in the HWs (and video for the Product Attribute HWs + Video participants) over the past week. Interpersonal discussion about HWs was measured by asking whether participants had talked to others about the messages and images in the warnings (and video for the Product Attribute HWs + Video participants) since viewing the warnings.

Smoke-limiting micro-behaviours comprised three individual items measured at follow-up. Participants were asked to report whether, over the past week, they had (i) tried to limit the number of cigarettes they smoked, (ii) stubbed or butted out a cigarette before finishing it, and (iii) stopped themselves from having a cigarette when they had the urge to smoke (‘yes’ or ‘no’/‘don’t know/can’t say’). The three micro-behaviours were examined as separate outcomes and as a composite variable measuring whether participants had engaged in at least one of the three smoke-limiting micro-behaviours in the past week.

#### Demographic characteristics and smoking variables

Participants reported their age, gender, highest level of education, whether they identified as Aboriginal or Torres Strait Islander, and as an indicator of socio-economic status, whether they held a health care card or pensioner concession card. Residential postcode determined regionality (metropolitan or regional) and socio-economic area using the Australian Bureau of Statistics 2021 Index for Relative Socio-economic Disadvantage (Australian Bureau of Statistics 2021).

Participants reported how many past-year quit attempts they had made, and their frequency of using FMC and RYO cigarettes, menthol and/or menthol ‘crushball’ (capsules in filters that are crushed to flavour the smoke) cigarettes, and e-cigarettes. Participants were classified as smoking predominantly menthol cigarettes if they smoked RYO and/or FMC menthol and/or menthol crushball products at least daily. If participants did not regularly smoke menthol cigarettes, or if the quota for predominant menthol smokers was met, participants were classified as predominantly smoking RYO or predominantly smoking FMC cigarettes, depending on which product they smoked more frequently.

### Statistical analysis

Data were analysed using Stata/MP version 16.1. Generalised linear regression models (Poisson) for binary outcomes and linear regression models for continuous outcomes were estimated to assess differences between the control and intervention conditions. For outcomes measured at baseline and follow-up (*changes in enjoyment* and *changes in concern*), time × condition interaction terms were included in the linear regression models. The beta coefficients computed from these interaction terms represent the estimated difference in rate of change between the control and intervention conditions. No models included covariates. All *P*-values computed from these models were adjusted for multiple comparisons using the Holm-Bonferroni method to balance the probability of Type I and Type II error. An alpha of 0.05 was used as the level of statistical significance.

To determine the effectiveness of the Product Attribute HWs, for each outcome we took the following steps: (i) compared responses among those exposed to Standard HWs to the control condition; (ii) compared responses among those exposed to Product Attribute HWs to the control condition; and then (iii), if either of the previous comparisons were significant at *P* < .05 after Holm-Bonferroni adjustment, we conducted post-estimation tests to compare the degree of difference between the control and each intervention condition (i.e. was the effect of exposure to Product Attribute HWs similar to the effect of exposure to Standard HWs). Similarly, to assess whether the effectiveness of Product Attribute HWs was enhanced by exposure to the complementary video, we (i) compared responses among those in the Product Attribute HWs condition to the control condition; (ii) compared responses among those in the Product Attribute HWs + Video condition to the control condition; and then (iii) if either of those comparisons were significant we conducted post-estimation tests to compare the degree of difference between the control and each of the Product Attribute HWs and Product Attribute HWs + Video conditions.

## RESULTS

### Participants

Table 1 shows the study sample characteristics at baseline (*N* = 2544) by condition. No clear evidence of differences between conditions in these characteristics was found, nor for the mean number of repeated exposure tasks completed [*P* > .05; control: *M* = 3.78 (Standard Deviation (SD) = 2.11); Standard HWs: *M* = 4.04 (SD = 2.05); Product Attribute HWs: *M* = 3.96 (SD = 2.01); Product Attribute HWs + Video: *M* = 3.86 (SD = 2.15); see Supplementary Table S4 for the distribution of repeated exposure tasks completed by condition].

**Table 1.** Socio-demographic and smoking characteristics of the study sample measured pre-exposure at baseline, by condition (*N* = 2544).

	Control	Standard HWs	Product Attribute HWs	Product Attribute HWs + Video	χ^2^ test
	*n* (%)	*n* (%)	*n* (%)	*n* (%)	*P*-value
Total	637 (100.0)	637 (100.0)	636 (100.0)	634 (100.0)	
Age group					.156
18–39 years	395 (62.0)	370 (58.1)	364 (57.2)	387 (61.0)	
40–54 years	182 (28.6)	179 (28.1)	198 (31.1)	183 (28.9)	
55–69 years	60 (9.4)	88 (13.8)	74 (11.6)	64 (10.1)	
Gender^a^					.133
Man/male	335 (52.6)	297 (46.6)	324 (50.9)	309 (48.7)	
Woman/female	298 (46.8)	339 (53.2)	309 (48.6)	323 (51.0)	
Another term	1 (0.2)	1 (0.2)	3 (0.5)	0 (0.0)	
Prefer not to say	3 (0.5)	0 (0.0)	0 (0.0)	2 (0.3)	
Highest level of education					.422
No tertiary education	358 (56.2)	372 (58.4)	348 (54.7)	363 (57.3)	
Tertiary education	274 (43.0)	261 (41.0)	281 (44.2)	270 (42.6)	
Socio-economic area					.197
Low	244 (38.3)	250 (39.3)	251 (39.5)	242 (38.2)	
Mid	275 (43.2)	274 (43.0)	240 (37.7)	254 (40.1)	
High	118 (18.5)	112 (17.6)	145 (22.8)	138 (21.8)	
Geographic region					.356
Metropolitan	475 (74.6)	454 (71.3)	448 (70.4)	463 (73.0)	
Regional	162 (25.4)	183 (28.7)	188 (29.6)	171 (27.0)	
Aboriginal and/or Torres Strait Islander^b^					.224
No	595 (93.4)	596 (93.6)	578 (90.9)	596 (94.0)	
Yes	36 (5.7)	34 (5.3)	48 (7.6)	32 (5.1)	
Prefer not to say	6 (0.9)	7 (1.1)	10 (1.6)	6 (1.0)	
Health care card or pensioner concession card holder^c^					.952
No	422 (66.3)	416 (65.3)	425 (66.8)	418 (65.9)	
Yes	215 (33.8)	221 (34.7)	211 (33.2)	216 (34.1)	
Quit attempts in past year					.410
None	297 (46.6)	290 (45.5)	300 (47.2)	291 (45.9)	
At least once	287 (45.1)	291 (45.7)	296 (46.5)	305 (48.1)	
Don’t know/can’t say	53 (8.3)	56 (8.8)	40 (6.3)	38 (6.0)	
Frequency of e-cigarette use					.852
Less than monthly	380 (59.7)	377 (59.2)	367 (57.7)	366 (57.7)	
At least monthly	257 (40.4)	260 (40.8)	269 (42.3)	268 (42.3)	
Frequency of FMC and/or RYO cigarette use					.319
Less than daily	128 (20.1)	150 (23.6)	138 (21.7)	152 (24.0)	
At least daily	509 (79.9)	487 (76.5)	498 (78.3)	482 (76.0)	
Predominant product use^d^					1.000
RYO cigarettes	130 (20.4)	133 (20.9)	132 (20.8)	130 (20.5)	
FMC cigarettes	357 (56.0)	354 (55.6)	347 (54.6)	353 (55.7)	
Menthol/menthol crushball RYO cigarettes	76 (11.9)	77 (12.1)	78 (12.3)	78 (12.3)	
Menthol/menthol crushball FMC cigarettes	74 (11.6)	73 (11.5)	79 (12.4)	73 (11.5)	

Abbreviations: HWs = health warnings; FMC = factory-made cigarettes; RYO = roll-your-own. Proportions are rounded so may not sum to 100.0%. No response was provided for the highest level of education for *n* = 5 in the control condition, *n* = 4 in the Standard HWs condition, *n* = 7 in the Product Attribute HWs condition, and *n* = 1 in the Product Attribute HWs + Video condition. Socio-economic area could not be computed for *n* = 1 in the Standard HWs condition.

^a^Chi-square test was conducted with ‘another term’ and ‘prefer not to say’ coded as missing due to limited observations.

^b^Chi-square test was conducted with ‘prefer not to say’ coded as ‘no’ due to limited observations.

^c^Individual-level measure of socio-economic status.

^d^RYO and FMC categories comprised both people who smoked menthol cigarettes and people who smoked non-menthol cigarettes.

A total of *N* = 1414 participants completed the follow-up survey (*N* = 1130 were not recontacted). This corresponded to a 55.6% retention rate overall, which did not differ by condition (control: 56.8%; Standard HWs: 53.4%; Product Attribute HWs: 56.0%; Product Attribute HWs + Video: 56.2%). A small number of participants did not consent to be recontacted for the follow-up survey (*n* = 197; 7.6%). Of the remaining *n* = 2347 participants that were eligible for follow-up, a multivariable model was conducted to identify any study characteristics (condition, number of repeated exposure tasks completed, additional outcomes measured immediately post-exposure), socio-demographic or smoking characteristics that significantly predicted attrition (Supplementary Table S5). This analysis indicated that women were more likely than men to complete the follow-up (56.7% vs. 43.4%; Prevalence Ratio (PR) = 1.14; 95% Confidence Interval (CI) = 1.08, 1.21; *P* < .001), those who had made a quit attempt in the past year were more likely than those who had not to complete the follow-up (48.9% vs. 43.9%; PR = 1.06; 95% CI = 1.00, 1.13; *P* = .039), and that the likelihood of participating in the follow-up increased as the number of repeated exposure tasks completed increased (PR = 1.24; 95% CI = 1.22, 1.26; *P* < .001).

There was no clear evidence of a difference between conditions in baseline socio-demographic characteristics among those who completed the follow-up survey (all *P*s > .05). There was also no evidence of a difference between conditions in the mean days between baseline and follow-up survey completion [*P* > .05; control: *M* = 10.46 (SD = 6.54); Standard HWs: *M* = 9.77 (SD = 4.51); Product Attribute HWs: *M* = 10.03 (SD = 5.46); Product Attribute HWs + Video: *M* = 9.72 (SD = 4.06)].

We then also examined the socio-demographic and smoking characteristics of the overall study samples at baseline and follow-up compared with Australian government population survey data (Supplementary Table S6). Compared to representative population data, the baseline and follow-up samples both tended to be younger, have a higher level of education, reside in higher socio-economic areas, and be more likely to report using e-cigarettes at least monthly, which likely reflects the rapid change in e-cigarette use behaviours in the years between population data collection (2019) (Australian Institute of Health and Welfare 2020) and the current study (2022). Consistent with the higher rate of attrition among males, the follow-up sample also contained an underrepresentation of men compared with the population data (Supplementary Table S6).

### Determining the effectiveness of Product Attribute HWs

**Figure 3.**
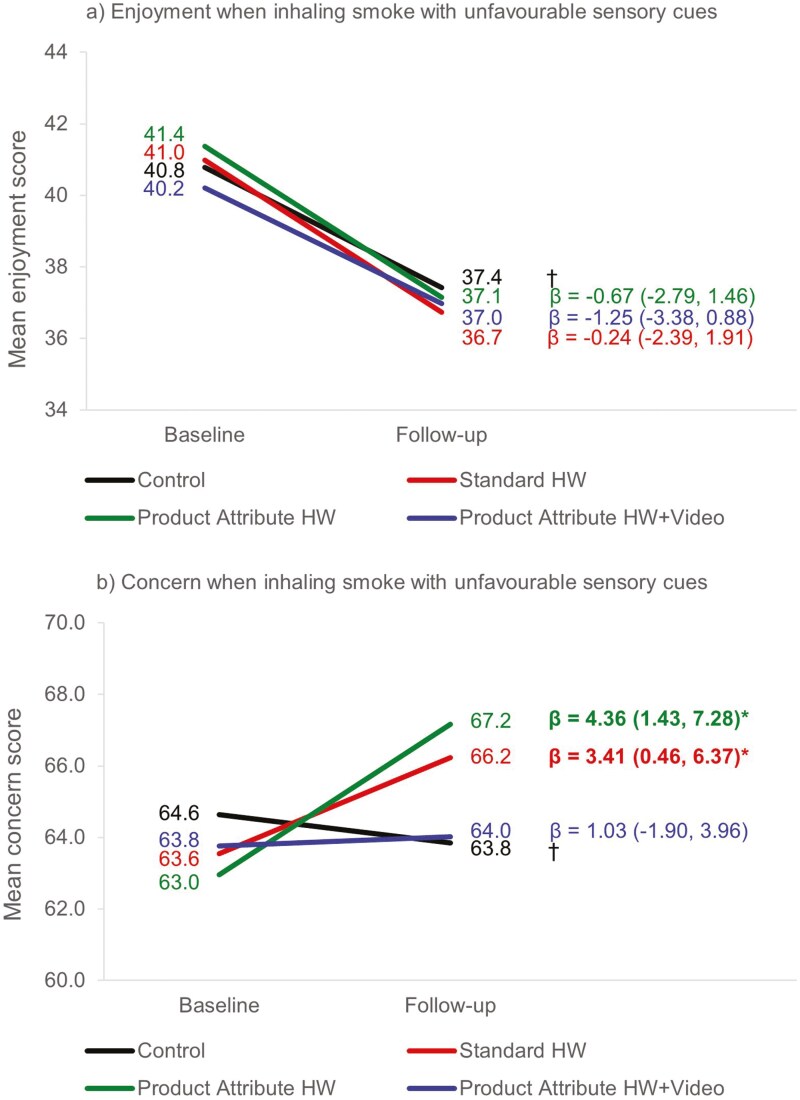
Change between baseline and follow-up in a) enjoyment and b) concern when inhaling smoke with unfavourable sensory cues, by condition. *Statistically significant difference at *P* < .05, compared to change in control condition (†). All *P*-values have been adjusted for multiple comparisons. Where significant *P*-values became non-significant after multiple comparison adjustment, this has been indicated (^). All regression models are unadjusted (i.e. no covariates included in the models).

Compared to control, participants in the Standard HWs condition had significantly higher scores on 5 of the 16 outcomes: change in concern when inhaling smoke with unfavourable sensory cues (*P* = .048; Fig. 3); self-centric negative emotional responses (*M* = 3.09 vs. 3.56, *P* < .001); industry-centric negative emotional responses (M = 3.00 vs. 3.24, *P* = .001); past-week rumination about warnings (16.0% vs. 29.7%, *P* < .001); and trying to limit the number of cigarettes smoked in the past week (60.0% vs. 70.9%, *P* = .006; Table 2).

**Table 2.** Primary outcomes at follow-up by condition and effect sizes for comparisons between Control and intervention conditions.

	Control^†^ *n* = 362	Standard HWs condition *n* = 340		Product Attribute HWs condition *n* = 356		Product Attribute HWs + Video condition *n* = 356		Standard HWs cf. Product Attribute HWs post-estimation comparison	Product Attribute HWs cf. Product Attribute HWs + Video post-estimation comparison
	Mean	Mean	β (95% CI)	Mean	β (95% CI)	Mean	β (95% CI)	*P*	*P*
Knowledge									
Knowledge of harms despite sensory cues	4.15	4.21	0.06 (−0.04, 0.16)	4.26	0.11 (0.01, 0.21)^	4.20	0.05 (−0.05, 0.15)	–	–
Knowledge of tobacco industry manipulation	3.97	3.99	0.02 (−0.09, 0.13)	4.10	0.13 (0.03, 0.24)*	4.09	0.12 (0.02, 0.23)*	.035*	.793
Negative emotional responses									
Self-centric negative emotional responses	3.09	3.56	0.47 (0.34, 0.60)***	3.52	0.43 (0.30, 0.56)***	3.48	0.38 (0.26, 0.51)***	.564	.503
Industry-centric negative emotional responses	3.00	3.24	0.24 (0.10, 0.38)**	3.42	0.42 (0.28, 0.56)***	3.44	0.44 (0.29, 0.58)***	.014*	.816
Product-specific smoking dissonance	3.25	3.39	0.14 (0.01, 0.26)^	3.42	0.16 (0.04, 0.28)*	3.34	0.09 (−0.03, 0.21)	.656	.248
	%	%	PR (95% CI)	%	PR (95% CI)	%	PR (95% CI)	*P*	*P*
Continued engagement with HWs									
Past-week rumination about HWs	16.0	29.7	1.85 (1.39, 2.47)***	31.7	1.98 (1.50, 2.62)***	34.0	2.12 (1.61, 2.80)***	.561	.524
Past-week online HW information-seeking	11.9	17.4	1.46 (1.01, 2.10)^	16.3	1.37 (0.95, 1.98)	21.1	1.77 (1.26, 2.51)**	–	.104
Past-week interpersonal discussion about HWs	19.1	25.6	1.34 (1.02, 1.77)^	24.2	1.27 (0.96, 1.68)	30.6	1.61 (1.23, 2.09)***	–	.055
Smoke-limiting micro behaviours									
Tried to limit the number of cigarettes smoked in past week	60.0	70.9	1.18 (1.06, 1.32)**	65.7	1.10 (0.98, 1.23)	64.0	1.07 (0.95, 1.20)	.145	–
Stubbed/butted out cigarette before finishing it in past week	56.6	62.6	1.11 (0.98, 1.25)	58.4	1.03 (0.91, 1.17)	64.6	1.14 (1.01, 1.28)^	–	–
Forgone a cigarette in past week	49.4	56.2	1.14 (0.99, 1.31)	53.9	1.09 (0.95, 1.26)	56.5	1.14 (0.99, 1.31)	–	–
At least one of the above smoke-limiting micro behaviours	79.8	86.2	1.08 (1.01, 1.15)^	80.6	1.01 (0.94, 1.09)	82.6	1.03 (0.96, 1.11)	–	–

Statistically significant difference at ***P < .001, **P < .010, and *P < .05. All *P*-values have been adjusted for multiple comparisons using the Holm-Bonferroni method, with the exception of post-estimation comparison *P*-values. Where significant *P*-values became non-significant after multiple comparison adjustment, this has been indicated (^). All regression models are unadjusted. ^†^Reference category. Abbreviations: HWs = health warnings; PR = prevalence ratio.

**Figure 4.**
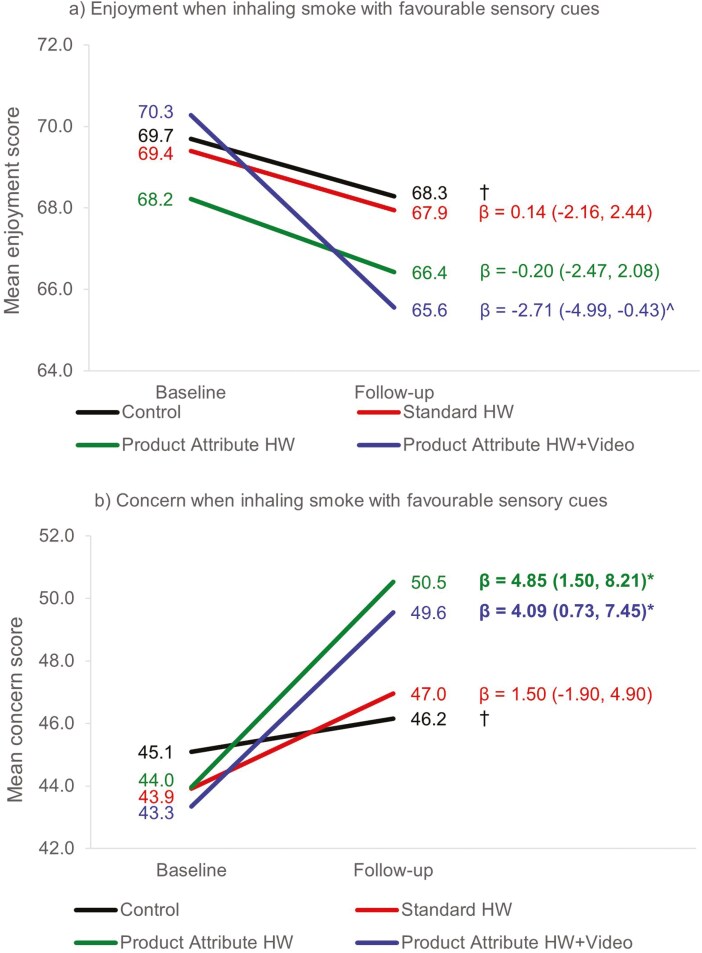
Change between baseline and follow-up in a) enjoyment and b) concern when inhaling smoke with favourable sensory cues, by condition. *Statistically significant difference at *P* < .05, compared to change in control condition (†). All *P*-values have been adjusted for multiple comparisons. Where significant *P*-values became non-significant after adjustment, this has been indicated (^). All regression models are unadjusted (i.e. no covariates included in the models).

Compared to control, participants in the Product Attribute HWs condition had significantly higher scores on 7 of the 16 outcomes: change in concern when inhaling smoke with favourable sensory cues (*P* = .015; Fig. 4); change in concern when inhaling smoke with unfavourable sensory cues (*P* = .012; Fig. 3); knowledge of tobacco industry manipulation (*M* = 3.97 vs. 4.10, *P* = .036); self-centric negative emotional responses (*M* = 3.09 vs. 3.52, *P* < .001); industry-centric negative emotional responses (*M* = 3.00 vs. 3.42, *P* < .001); product-specific smoking dissonance (*M* = 3.25 vs. 3.42, *P* = .027); and past-week rumination about HWs (16.0% vs. 31.7%, *P* < .001; Table 2).

Post-estimation tests indicated that the effect in the Product Attribute HWs condition (vs. control) was significantly larger than the effect in the Standard HWs condition (vs. control) for knowledge of tobacco industry manipulation (*P* = .035) and industry-centric negative emotional responses (*P* = .014). The effect in the Product Attribute HWs condition (vs. control) was larger than the effect in the Standard HWs condition (vs. control) for change in concern when inhaling smoke with favourable sensory cues although this difference only approached statistical significance (*P* = .053), while the effects of the two conditions were of a similar magnitude for change in concern when inhaling smoke with unfavourable sensory cues (*P* = .533), self-centric negative emotional responses (*P* = .564), product-specific smoking dissonance (*P* = .656), past-week rumination about HWs (*P* = .561), and trying to limit the number of cigarettes smoked in the past week (*P* = .145; Table 2).

### Determining the effectiveness of Product Attribute HWs + Video

Compared to control, participants in the Product Attribute HWs + Video condition had significantly higher scores on 7 of the 16 outcomes: change in concern when inhaling smoke with favourable sensory cues (*P* = .034; Fig. 4); knowledge of tobacco industry manipulation (*M* = 3.97 vs. 4.09, *P* = .048); self-centric negative emotional responses (*M* = 3.09 vs. 3.48, *P* < .001); industry-centric negative emotional responses (*M* = 3.00 vs. 3.44, *P* < .001); past-week rumination about warnings (16.0% vs. 34.0%, *P* < .001); past-week online warning information-seeking (11.9% vs. 21.1%, *P* = .003); and past-week interpersonal discussion about warnings (19.1% vs. 30.6%, *P* < .001; Table 2).

Post-estimation tests indicated that the effect in the Product Attribute HWs + Video condition (vs. control) was significantly *smaller* than the effect in the Product Attribute HWs condition (vs. control) for change in concern when inhaling smoke with unfavourable sensory cues (*P* = .026; Fig. 3). The effect in the Product Attribute HWs + Video condition (vs. control) was *greater* than the effect in the Product Attribute HWs condition (vs. control) for past-week interpersonal discussion about HWs, although this difference was only approaching statistical significance (*P* = .055), while the effects of the two conditions were of a similar magnitude for change in concern when inhaling smoke with favourable sensory cues (*P* = .656), knowledge of tobacco industry manipulation (*P* = .793), self-centric negative emotional responses (*P* = .503), industry-centric negative emotional responses (*P* = .816), product-specific smoking dissonance (*P* = .248), past-week rumination about HWs (*P* = .524), and past-week online HW information-seeking (*P* = .104; Table 2).

### Sensitivity analysis

We undertook analyses of the sub-group of participants who predominantly smoked FMC cigarettes (*N* = 1411 at baseline; *N* = 782 at follow-up), among whom no clear evidence of differences in socio-demographic characteristics was observed between conditions. Findings were generally consistent with the full sample results (see Supplementary Table S7).

In the full sample, our participants had a higher level of education than the general population of people who smoke, leading to a potential concern that our sample may have been more receptive to the arguments in our Product Attribute HWs. We therefore limited analyses to participants with no tertiary education (*N* = 1441 at baseline; *N* = 861 at follow-up), among whom no clear evidence of differences in sociodemographic characteristics was observed between conditions. Findings were again largely consistent with the full sample results (see Supplementary Table S8).

Finally, four of the outcomes measured at follow-up were also measured immediately post-exposure during the baseline survey: knowledge of harms despite sensory cues; knowledge of tobacco industry manipulation; self-centric negative emotional responses; and industry-centric negative emotional responses. Due to the high rate of attrition, we examined whether the pattern of responses was similar for the immediate post-exposure and follow-up versions of these variables (see Supplementary Table S9). Effects were typically larger immediately post-exposure, however, these sensitivity analyses reassured us that attrition biases were unlikely to be having a large effect on results for these four outcomes measured at follow-up.

## DISCUSSION

In this study, exposure to the new Product Attribute HWs had beneficial short-term effects and increased knowledge of tobacco product manipulations, feelings of concern, other negative emotional responses, and feelings of discord and discomfort when smoking. However, there was no evidence that exposure to the Product Attribute HWs increased smoke-limiting behaviours. Specifically, compared to the control, those in the Product Attribute HWs condition reported greater increases in concern when inhaling smoke with misleading favourable sensory cues (e.g. smooth) and unfavourable sensory cues (e.g. harsh), more knowledge of industry product manipulation, greater self-centric negative emotional responses (felt uncomfortable, worried and embarrassed), greater industry-centric negative emotional responses (felt angry at and deceived by industry), higher levels of dissonance about smoking, and a higher likelihood of past-week rumination about warnings. Importantly, as discussed further below, some effects observed for the Product Attribute HWs were similar to those for the Standard HWs, while others were unique to the Product Attribute HWs. This finding suggests that Product Attribute HWs may have an important and complementary role to play as part of a suite of diverse HWs that includes messages focussed on health effects of smoking alongside messages about product manipulations.

Many outcomes were similar for those exposed in the Standard HWs and Product Attribute HWs conditions. Both had similarly elevated change over time in concern about inhaling smoke with unfavourable sensory cues, and greater self-centric negative emotional responses to and past-week rumination about HWs, indicating similar emotional and cognitive engagement. Neither the Standard HWs nor Product Attribute HWs changed enjoyment when inhaling smoke with favourable or unfavourable sensory cues. This finding suggests that enjoyment is a more difficult outcome to influence, perhaps because it may be tied to the pervasive underlying drive for nicotine-induced satisfaction. Neither HW type resulted in greater knowledge about harms despite sensory cues, although we note that scores were high, even in the control group (4.15 of a 5.0 maximum), suggesting further increases would be challenging. Neither HW type resulted in more foregoing or butting out of cigarettes, although Standard HWs were effective in prompting participants to try to limit the number of cigarettes smoked. Notably though, the post-estimation comparison indicated that there were comparable effect sizes for the Standard HWs condition and Product Attribute HWs condition on eliciting efforts to limit the number of cigarettes smoked, suggesting that both types of HWs may be similarly effective at encouraging this behaviour.

Participants in the Product Attribute HWs condition uniquely reported greater knowledge of tobacco industry manipulation, consistent with product HW message specificity. While participants in both the Standard HWs and Product Attribute HWs conditions reported greater industry-centric negative emotional responses than the controls, this effect was greater among Product Attribute HWs participants. Importantly, secondary analyses from this study indicated that the novel industry-centric outcomes that were uniquely affected by the Product Attribute HWs (knowledge of tobacco industry manipulation, and industry-centric negative emotional responses) predict subsequent changes in downstream quitting-related behaviours, confirming the importance of these proximal measures (Brennan et al. 2024). Consistently, previous research has indicated that informing people about the mechanisms of various product manipulations is important because, aside from the persuasive benefits of imparting such explicit information (Cook and Lewandowsky 2020), holding more negative views about the tobacco industry increases quit intentions (Lee et al. 2019, Nguyen et al. 2019) and contributes to industry denormalisation (Malone et al. 2012), which itself reduces smoking prevalence (Malone et al. 2012).

Those in both the Product Attribute HWs and Product Attribute HWs + Video conditions had similarly elevated knowledge of tobacco industry manipulation, self- and industry-centric negative emotional responses, and rumination about HWs. Only those in the Product Attribute HWs + Video condition reported greater inter-personal discussion and online information-seeking about HWs compared to the control, behaviours that predict quit attempts (Jeong et al. 2015, Thrasher et al. 2016, Morgan et al. 2018). Packs and pouches provide limited space for communicating corrective information and are static, while multi-media campaigns can make complex information clearer and more compelling. In addition, government-mandated pack warnings may be unable to explicitly call out the tobacco industry, whereas public communication campaigns run by or partnering with non-government health organizations could do so. Our findings are consistent with other research that suggests comprehensive campaigns can extend and amplify the positive outcomes of HWs in populations (Brennan et al. 2011, Thrasher et al. 2013, Nagelhout et al. 2015).

### Strengths and limitations

A strength of the study was that we addressed three common industry product manipulations of filter-venting, menthol, and RYO tobacco that promote consumer misperceptions, as well as clarifying that most of the harm comes from combustion, not additives. The Product Attribute HWs therefore target all people who smoke and communicate a meta-message that no-one can escape harm by smoking tobacco with particular product features or sensory cues.

Second, we used a conceptual model to develop the Product Attribute HWs which were pre-tested iteratively in focus groups and quantitative studies, confirming that each Product Attribute HW achieved perceived effectiveness criteria and improved relevant understanding. Third, we compared Product Attribute HWs to a non-tobacco warning control group and a set of refreshed Standard HWs with new serious smoking-related harms and new information about familiar harms using a comparable format to Product Attribute HWs. This approach constituted a more rigorous test of the Product Attribute HWs and minimized differences in information novelty between Standard HWs and Product Attribute HWs. Fourth, our study design included the potential for repeated exposure over 7 days to better simulate a real-world pattern of HW exposure. Not all participants opted to be re-exposed, which reflects variation in HW and video engagement in everyday life.

The most notable limitation was that the study was somewhat under-powered. The retention rate of 55.6% was lower than the expected 64% (based on the panel provider’s experience with similar studies) and may have missed some real effects; our reported differences in outcomes between conditions are thus conservative. Relatedly, the prevalence of forgoing a cigarette, on which primary power calculations for Product Attribute HWs were based, was lower than expected in every condition, perhaps reflecting different years of data collection (2019–2021 for the reference study (Thrasher et al. 2024) vs. 2022 for the current study) and different country contextual factors, as well as different methods of data collection. The prevalence of any one of three smoke-limiting micro-behaviours, on which power calculations for the Product Attribute HWs + Video comparison were based, was 10%-points higher in the control group than expected, again potentially due to different contextual factors between the reference study (Unpublished data: Mitsopoulos, Vittiglia, & Durkin, 2021. *Outcome evaluation of the Sticky Blood campaign (2021)*. Centre for Behavioural Research in Cancer, Cancer Council Victoria. Melbourne, Australia) and the current study.

We observed a higher rate of attrition among males than females, and consequently, males were under-represented in the follow-up sample compared with what we expected based on population data. Furthermore, our baseline and follow-up samples both tended to be younger, be more highly educated, reside in higher socio-economic areas, and be more likely to report using e-cigarettes at least monthly than the population sample. Online non-probability panels do not provide a random population sample, and so we do not suggest our parameter estimates statistically represent the national population of people who smoke. On the other hand, we quota-sampled to ensure we recruited sufficient participants who smoked menthol cigarettes to the study, and this may partly explain other differences between our study sample and population data. Additionally, we observed no differential attrition by condition, so internal validity was high. Finally, while a potential limitation was a follow-up of only 8 days, most primary outcomes were established precursors of later change in quit intention or attempts (Partos et al. 2014, Jeong et al. 2015, Li et al. 2015, Thrasher et al. 2016, 2019, Cho et al. 2018, Morgan et al. 2018, Brewer et al. 2019, Lee et al. 2019, Nguyen et al. 2019, Lambert et al. 2020).

### Future research

Future studies could assess the impact of exposure to a set of Standard HWs compared to a set of Standard HWs and Product Attribute HWs combined since, in practice, Product Attribute HWs would be likely to be implemented alongside Standard HWs. While our study design enabled us to identify outcomes that were specific to Product Attribute HWs, this alternative design would test the aggregate effects of exposure to Standard HWs and Product Attribute HWs. Studies might also assess the extent to which people become more supportive of banning misleading cigarette manipulations after exposure to Product Attribute HW messages and videos.

## CONCLUSION

Using Product Attribute HWs that address misperceptions about filter-ventilation, menthol, RYO tobacco, and combustion yields many similar positive outcomes to Standard HWs, but also offer unique beneficial outcomes. Thus, Product Attribute HWs do not substitute for Standard HWs but complement them. Because Product Attribute HWs can challenge long-standing myths about tobacco products fostered by tobacco companies, nations should look to include Product Attribute HWs in their suites of tobacco HWs.

## Supplementary Material

daae210_suppl_Supplementary_File1

daae210_suppl_Supplementary_Tables_S1-S9

## Data Availability

The data underlying this article will be shared upon reasonable request to the corresponding author.
