# Supplementary material for: Tobacco package health warnings about product manipulations: an experimental study among Australian adults who smoke
Source: Health Promot Int. 2025 Apr 3;40(2):daae210. doi: 10.1093/heapro/daae210 (PMC11965984; doi:10.1093/heapro/daae210)
Supplement: daae210_suppl_Supplementary_File1 [file daae210_suppl_supplementary_file1.docx]

**SUPPLEMENTARY FILE: Section 1**

**Preliminary and pre-testing studies to develop Product Attribute Health Warnings**

**Study 1a: Preliminary focus groups of roll-your own (RYO) smokers**

Objective: To explore smoking beliefs and experiences of those who predominantly smoke RYO tobacco.

Method: We conducted 10 focus groups in June 2018 of adults who predominantly smoked RYO tobacco, segmented by age, sex and education, with a total of 65 RYO smokers aged 18 to 39. We used thematic analysis to interpret the transcripts.

Results: Price was a substantial motivator for RYO use, likely due to recent annual tax increases substantially raising the expense of smoking. Participants valued the availability of smaller sized pouches (<30 grams, with some pouches weighing just 15 grams) as these provided a cheaper upfront cost and prevented the tobacco from drying out as happens with larger pouches. They typically perceived RYO tobacco as higher quality than tailor-made cigarettes, although in some cases this association appeared to rationalise their use of ‘cheap’ tobacco. This perception of higher quality was driven by smokers’ perceptions of RYO’s smoother taste and ‘mouthfeel’ compared to the harsher experience of smoking tailor-made cigarettes, observation of RYO’s moistness, and assumptions about RYO being more natural. While many RYO smokers acknowledged that smoking either product was likely harmful to health, some still regarded RYO tobacco as less harmful than tailor-made cigarettes or, at least, hoped this was the case.

Conclusions/ Implications: As found in other nations, lower price was the primary motivator for RYO tobacco use among Australian smokers. Other perceived benefits, such as quality, taste, naturalness, and reduced harm, contribute to RYO smokers’ preference for RYO tobacco.  Mass media campaigns and pouch warnings could correct misperceptions that RYO tobacco is less harmful than tailor-made cigarettes.

Note: Results of this study were presented at the Australian Behavioural Research in Cancer Conference, Perth, Australia in 2019.

**Study 1b: Preliminary focus groups of people who smoke tailor-made (TM) cigarettes**

Objective: To explore the extent to which TM smokers were aware of filter-venting, how they describe how the sensory experience of smoke varies, and their beliefs about related harms.

Method: We conducted eight focus groups in late 2018 with TM smokers aged 20 to 55 years, segmented by age, sex and education. We used thematic analysis to interpret the transcripts.

Results: Red variants (least ventilated) were typically described as harsh, heavy and strong, with more smoke in each inhalation. The smoke from Gold variants (more ventilation) was generally described as smooth, light and weak. Blue variants were regarded as an acceptable medium, although Blues were believed to be closer to Golds in strength than Reds. Inhaling the smoke from Gold variants felt less immediately damaging to the lungs, while smoke from Red variants was irritating and described as more obviously harmful to participants. Smokers were generally unsure why some cigarettes felt smooth and few were aware of filter-ventilation. Participants then read a short explanation of filter-ventilation, its actions and consequences. This was largely perceived as new information and some valued the explanation as speaking to them as adults rather than trying to scare them. Some developed an understanding that the manufacturing process was dictating their smoking experience. Some participants were quite upset at the thought they had been manipulated into believing that variants producing lighter or smoother smoke were not as harmful as variants that produced harsher smoke. Finally, some were more defensive, noting that they already knew smoking was harmful and asserting their desire to smoke was theirs alone.

Conclusions/ Implications: There is a need to educate smokers about filter-venting. The varied reactions suggest this information has the capacity to at least raise questions for some about their smoking and to elicit industry-directed concern and anger among others.​ Consumer and public education might usefully reframe the experienced lightness and smoothness of cigarette smoke as being the result of tobacco industry manipulation to mask the underlying harm.​

Note: Results of this study were presented at the Oceania Tobacco Control Conference in Sydney, Australia in 2019.

**Study 2: National population survey of adults who smoke**

Objective: To determine common misperceptions among people who smoke tailor-made (TM) cigarettes, RYO tobacco, and menthol cigarettes, with a view to assisting the construction of mental models that underpin product and harm misperceptions.

Method: In July 2019, we undertook a national survey of 999 people who smoked tobacco at least weekly, recruited from an online non-probability panel. The survey measured the extent to which smokers associate certain sensory experiences with smoking cigarettes that are: menthol vs non-menthol (e.g. fresh, cooling, soothing, etc); TM vs RYO (e.g. moist, stale, natural etc); and within TM cigarettes, those of differing colour varieties (in other words, more vs less highly ventilated) in terms of smoke harshness, smoothness, lightness etc. We also investigated their beliefs about how enjoyable and how damaging they found cigarettes with each of these sensory experiences. The survey also sought to establish the prevalence within people who smoke of smoking different types of tobacco products, including colour varieties, and cigarettes with different types of filters such as firm filters and recessed filters, and beliefs relevant to these.

Results: In relation to filter-venting, TM smokers (n=418) were significantly more likely to report that cigarettes with more filter venting (e.g. gold and blue variants) felt smooth, weak and light, than cigarettes with fewer vents (e.g. red variants). Furthermore, smokers who experienced gold / blue cigarettes as smooth and light were significantly more likely to mistakenly believe those cigarettes are less damaging. In relation to RYO tobacco, people who exclusively smoked RYO (n=157) had almost eight times the odds of experiencing RYO cigarettes than TM cigarettes as being moist vs. dry and having a natural vs. chemical taste.  Cigarettes with moist and natural tasting tobacco were commonly perceived as being more enjoyable and less damaging.  In relation to menthol, menthol smokers (n=252) were four to nine times more likely to experience menthol rather than non-menthol cigarettes as having favourable sensory experiences, including feeling smooth, being soothing on the throat, fresh tasting, and clean feeling. Menthol smokers perceived cigarettes with these favourable sensations as less damaging and more enjoyable than cigarettes with the opposite more aversive sensory experience.

Conclusions/ Implications: The survey confirmed that the sensory experience of smoking particular tobacco products plays a key role not only in increasing smoking enjoyment, but in driving misperceptions about smoking harm. Building upon the results of prior focus groups, the survey findings also allowed us to focus most in subsequent corrective communication development on the terms to describe sensory experiences that were most strongly related to harm misperceptions.

Note: Results pertaining to TM colour variants were presented at the Oceania Tobacco Control Conference in Sydney, Australia in 2019. Results have been published from the n=999 participants pertaining to the prevalence of smoking cigarettes with recessed and/or firm filters and related beliefs,^[[1]](#footnote-2)^ and the sensory experiences and beliefs of the n=252 people who smoke menthol cigarettes.^[[2]](#footnote-3)^

**Study 3: Explanatory text message development**

Objective: In Australia, explanatory text statements are included on the back of the pack/pouch to provide further detail about the health warning described in the warning statement on the front of the pack/pouch. This study aimed to determine the effectiveness among smokers of different ways of explaining the misleading sensory experiences elicited by different tobacco product attributes (filter-venting, RYO tobacco, menthol cigarettes) and why these sensory experiences are of concern.

Method: In July 2020, we undertook a national online pre-testing study of potential explanatory statements involving 554 people aged 18 to 69 years who smoked TM, RYO or menthol cigarettes at least weekly. Participants who smoked Red variety cigarettes (which are the least ventilated) were excluded from the study. Following Lewandowsky and colleagues’ conceptual framework for effectively correcting misperceptions,^[[3]](#footnote-4)^ each explanatory statement comprised three elements: (a) acknowledges a sensory experience that smokers associate with the product (e.g. ‘*Menthol makes cigarettes taste fresh. It also triggers a feeling of cold in your mouth and throat.’*); (b) links it with a new core fact to help smokers reframe the meaning of that sensory experience (e.g. ‘*The cold feeling hides the burning sensation of toxic smoke.’*) and (c) offers a ‘so what’ message that provides a reason for smokers to care about this new information (e.g. ‘*This means you may not notice the damage being done to cells in your mouth and lungs’*). We prepared 48 potential explanatory text statements (without images) using findings from Studies 1 and 2. Some explanatory statements were relevant to all smoker groups, while others were tailored to TM smokers (addressing two sensory experiences and five core facts), RYO smokers (addressing three sensory experiences and five core facts) or menthol smokers (addressing three sensory experiences and six core facts). Half of these explanatory statements contained explicit mention of tobacco industry involvement (e.g. ‘*Tobacco companies put profits before smokers’ health.’)* while half did not. Participants were assigned to one of three conditions—menthol (n=140), RYO (n=140) or TM cigarettes (n=140)—depending on the products that each participant smoked at least weekly; they therefore viewed explanatory statements of most relevance to their smoked products. Each participant viewed 6 to 7 statements, providing a total of around 35 ratings per statement. After viewing each statement, participants completed standard perceived effectiveness (PE) ratings and then were debriefed on the study.

Results: Overall, explanatory statements performed well on PE scores, although PE scores varied for statements pertaining to different sensory experiences and core facts. There were no differences between PE ratings for explanatory statements that did or did not explicitly mention tobacco industry involvement, including on the outcome of feeling deceived by the tobacco industry.

Conclusions/ Implications: The PE scores helped us short-list a total of 14 explanatory statements for further testing, including four statements for lighter TM smoke, three statements for RYO tobacco, four statements for menthol cigarettes and three statements for smooth smoke (which applied to all three product types). In relation to our finding on explicitly mentioning tobacco industry involvement (or not), we hypothesized that Australian smokers already have low trust in the tobacco industry, such that the messages which are more implicit about tobacco industry creation of misleading sensory experiences are sufficient for eliciting perceptions of industry deception, while also achieving the other PE outcomes. Explanatory messages that do not explicitly call out tobacco industry involvement may be most appropriate for government-endorsed health warnings on tobacco packs/pouches. However, non-government organisations and collaborative public communication campaigns who have more freedom in messaging approaches could still take the approach of more explicitly calling out the tobacco industry.

Note: The findings for mentions of tobacco industry involvement in menthol cigarettes were presented at the Australian Preventive Health Conference in May 2021.

**Study 4: Focus group pre-test of Product Attribute Health Warning (PAHW) headline statements, large pictorial images and explanatory texts**

Objective: To explore smokers’ comprehension, understanding and preferences for different pictorial image and headline combinations which accompany a short-listed set of explanatory statements for PAHWs.

Method: We conducted 14 online focus groups in September/October 2020, segmented by age (18-25, 26-44, 45-69), sex (for the younger group) and product type (TM, RYO, menthol), with a total of 84 smokers aged 18 to 69 years skewed towards lower educational attainment who smoke at least 10 cigarettes of TM, RYO or menthol per week. Each focus group viewed and discussed six to seven explanatory text statements, each with two to three alternative warning statements and associated images. Thematic analysis was used to interpret the transcripts.

Results: The information presented was generally assessed by participants as new, informative and engaging. Pleasingly, it increased participants’ understanding that the manufacturing process was exploiting their smoking preferences. Messages about menthol were clearer to participants than messages about filter-venting and RYO tobacco. An unintended finding was that participants (mistakenly) thought that additives could be contributing *more* to the dangers of smoking than they had previously been aware of. Relatively small suggestions were made to improve clarity of headlines, images and text of particular PAHWs.

Conclusions/ Implications: A revised set of PAHWs needs to include at least one discrete warning that provides clear foundational information for all smokers that most of the harm of inhaled tobacco smoke comes from the process of combustion, and not from additives, and to reinforce that additives are mostly included to mask the harshness of smoke so that it can be more easily inhaled.

Note: Focus group testing results for corrective RYO warnings with RYO smokers were presented at the Australian Preventive Health Conference in 2021.

**Study 5: Focus group pre-test of revised and extended Product Attribute Health Warning (PAHW) headline statements, large pictorial images and explanatory texts**

Objective: To explore smokers’ comprehension, understanding and preferences for different pictorial image and headline combinations which accompany a revised set of explanatory statements for PAHWs about filter-venting and RYO tobacco and new PAHWs pertaining to combustion as the main source of harm rather than additives.

Method: We conducted 10 online focus groups in February 2021, segmented by age (18-25, 26-44, 45-69), sex (for the younger group) and product type (TM, RYO). The groups comprised a total of 51 smokers aged 18 to 69 years skewed towards lower educational attainment who smoked at least 10 cigarettes of TM or RYO per week. Each focus group viewed and discussed six to seven explanatory text statements, each with two to three alternative warning statements and associated images. Thematic analysis was used to interpret the transcripts.

Results: Participants again appreciated the novelty of these revised and extended PAHWs and easily engaged with them. The overall message take-out was that all cigarettes and tobacco are harmful no matter whether they are light, smooth or RYO. Even though messages did not explicitly call out the tobacco industry, participants identified that factors associated with the tobacco manufacturing process change the smoking experience, and specifically that substances added during the process reduce the unpleasantness they would experience from smoking less processed tobacco. This realisation left participants feeling that they had been manipulated by the industry. Information about the manufacturing process was perceived as factual or objective and hard to dismiss. Participants expressed interest in finding out more about the product, perhaps via URL or QR Codes on packs/pouches. Relatively minor tweaks were suggested to improve message clarity.

Conclusions/ Implications: Overall, the PAHWs performed as desired and the new combustion warning performed well in clarifying where most harm came from. While minor revisions were needed to some PAHWs, most were ready for quantitative pre-testing.

**Study 6: Quantitative pre-testing study of complete PAHWs and THWs**

Objective: To assess smokers’ ratings of the potential effectiveness of Product Attribute Health Warnings (PAHWs), and to assess the extent to which PAHWs corrected specific misperceptions. For comparative purposes, we also assessed the potential effectiveness of 14 Tobacco Health Warnings (THWs) that were designed to be used in the THW arm of the experimental study. These THWs had been developed by collaborating with a team with expertise in tobacco warning labels and science communication and were in a format similar to those proposed for the new Australian THWs.

Method: In June 2021, we conducted a cross-sectional online message rating study of at least weekly smokers aged 18 to 69 years of non-menthol tailor-made (TM) cigarettes, non-menthol roll-your-own (RYO) cigarettes or menthol TM and/or RYO cigarettes. Participants were allocated according to their predominant product use to one of four study arms: the TM cigarette arm (n=97), the RYO cigarette arm (n=95), or the menthol cigarette arm (n=92). Additional participants (n=132) were assigned to the THW arm (n=132) which included a mix of menthol/non-menthol TM and RYO smokers. Each PAHW and THW comprised: (a) a headline and photographic or digitally produced image on the front of pack/pouch; (b) an explanatory statement, another image, and in some cases a secondary headline on the back of pack/pouch; and (c) a written message on the side of pack (TM) or under flap (RYO). A consistent colour scheme was used for all PAHWs/THWs. Smokers were first shown the front of pack/pouch, then the back of pack/pouch, and then side of pack/under flap views, before being shown an image of the full PAHW or THW with all components and asked to rate it on perceived effectiveness (PE) measures. All PAHWs/THWs were displayed to participants on either tailor-made packs or RYO pouches, depending upon whether the participant predominantly smoked TM or RYO. Participants in the three PAHW arms viewed and rated a random order of six PAHWs that were relevant to the product they smoked. In the THW arm, participants viewed and rated a random selection of seven of 14 THWs. Finally, all participants were presented with a series of statement pairs that either stated a product attribute misperception or its correction and asked to choose which statement in each pair was correct. Participants could respond don’t know/can’t say. They then answered additional demographic questions and were debriefed about the purpose of the study.

Results: To confirm comparability between PAHW and THW sets, we examined the mean PE scores for each outcome across the whole set of PAHWs and THWs.​ While there was variation within PAHWs and THWs, all PAHWs and THWs scored well on ten potential effectiveness outcomes. Retaining the top 11 best-performing THWs while preserving most similarity in health harms mentioned in the THWs and PAHWs resulted in similar scores across PE outcomes (see Figure 1). The sole exception was that PAHWs attained higher scores than THWs on ‘This message made me angry at tobacco companies’, which was an implicit message of the PAHWs but not the THWs and thereby provided good discriminant validity. Finally, for 10 of 11 statement pairs that either restated a PAHW misperception or provided a correct statement about that topic, TM/RYO/menthol smokers exposed to their respective PAHWs were statistically more likely to select the correct statement pair than the relevant comparable subset of TM/RYO/menthol smokers exposed only to the THWs (see Figure 2). For the remaining statement pair, the % correct among PAHW participants was at least as good as for THW participants.

Figure 1: Mean PE scores (% agree) by outcome for PAHWs and THWs


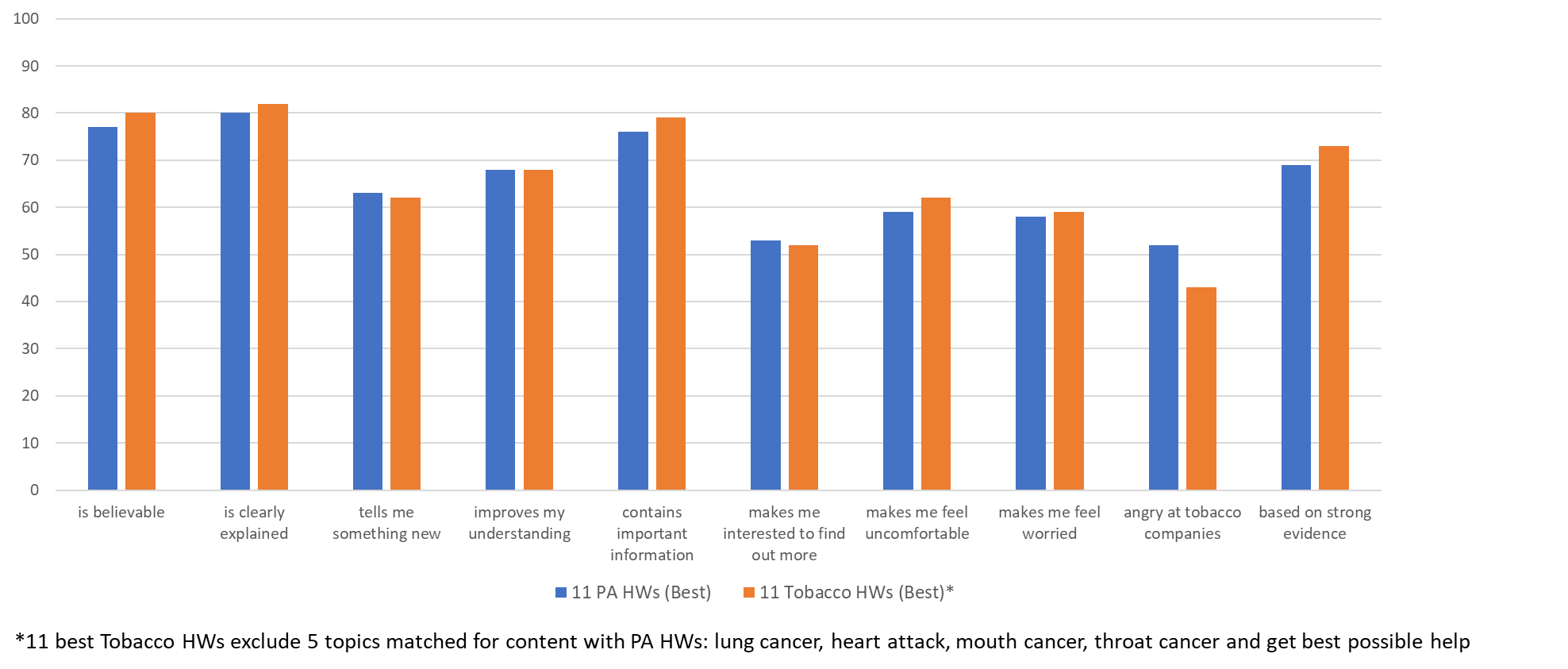


Figure 2: % who selected the correct statement among comparable sub-sets of tobacco product users exposed to either PAHWs or THWs


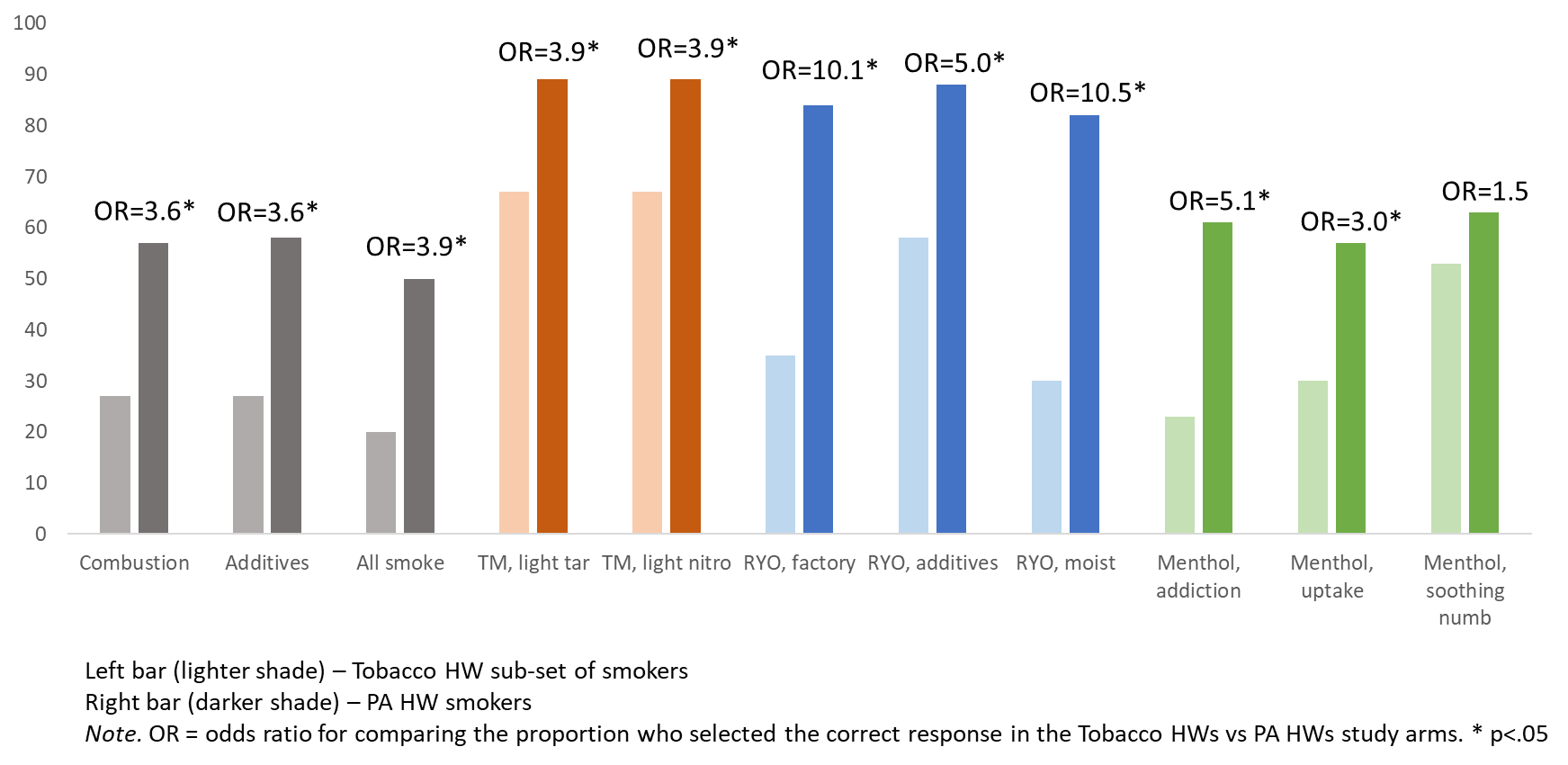


Conclusions/Implications: This study confirmed that our set of 11 developed PAHWs was potentially effective as measured by PE scores. It enabled us to select the most comparable subset of 11 THWs in terms of PE scores and type of health harms mentioned for inclusion in the experimental arms of the main experimental study. Finally, we confirmed that nearly all our 11 individual PAHWs corrected misperceptions about the specific product attribute they addressed. Reflecting that health warnings need to operate in practice more as a team or a set rather than as individual warnings, we therefore proceeded to use all 11 PAHWs within our set of PAHWs for the main study.

Note: We presented at the 2022 Australian Preventive Health Conference and the 2022 Society for Research on Nicotine and Tobacco Conference on our qualitative and quantitative research to develop PAHWs on filter-ventilation, mentioning Study 3 (explanatory text PE study), Study 5 (focus group testing of revised headline text, images and explanatory texts) and Study 6 (quantitative pre-testing of complete PAHWs).

1. Wakefield M, Dunstone K, Brennan E, Vittiglia A, Scollo M, Durkin SJ, Hoek J, Thrasher J, Hatsukami D, Benowitz N, Samet JM. Australian smokers’ experiences an perception of recessed and form filter cigarettes. *Tobacco Control* 2020; Nov;30(6):660-667. doi:10.1136/tobaccocontrol-2020-055725.  [↑](#footnote-ref-2)
2. Mancuso S, Brennan E, Dunstone K, Vittiglia A, Durkin S, Thrasher JF, Hoek J, Wakefield M. Australian smokers sensory experiences and beliefs associated with menthol and non-menthol cigarettes. *Int J Envir Res Public Health* 2021; May 21;18(11):5501. doi:10.3390/ijerph18115501. [↑](#footnote-ref-3)
3. Lewandowsky S, Ecker UK, Seifert CM, Schwarz N, Cook J. Misinformation and its correction: Continued influence and successful debiasing. *Psychological Science in the Public Interest* 2012; 13(3):  106-31. [↑](#footnote-ref-4)
