# Supplementary material for: Tobacco package health warnings about product manipulations: an experimental study among Australian adults who smoke
Source: Health Promot Int. 2025 Apr 3;40(2):daae210. doi: 10.1093/heapro/daae210 (PMC11965984; doi:10.1093/heapro/daae210)
Supplement: daae210_suppl_Supplementary_Tables_S1-S9 [file daae210_suppl_supplementary_tables_s1-s9.docx]

**SUPPLEMENTARY FILES: Table S1 to Table S8**

Table S1. Text included on the front, side and back of the 11 Product Attribute Health Warnings.

| **Front-of-pack/pouch** | **Side-of-pack/pouch** | **Back-of-pack/pouch** |
| --- | --- | --- |
| Combustion creates new toxins | | |
| **Harmful chemicals are created when tobacco is burned.** | If a cigarette is burning, it’s creating new toxic chemicals. These chemicals get into your cells, where they damage your DNA.  You CAN quit smoking. Call Quitline 13 7848 | **Cigarette smoke contains hundreds of toxic chemicals.**  Some of these are in the living tobacco plant.  Some are formed when the tobacco plant is dried and cured.  Some come from additives used as the tobacco is being processed.  But when you light your cigarette, it starts a chemical reaction called combustion.  Combustion of the tobacco creates new toxic chemicals that cause MOST of the harm. |
| Additives hide true harshness | | |
| **Additives hide the smoke’s true harshness.** | When additives hide the harshness of the smoke, it’s easier for you to inhale the chemicals in the smoke that cause cancer.  You CAN quit smoking. Call Quitline 13 7848 | **Additives are used to make your cigarettes easier to smoke.**  Without additives, smoke from a burning cigarette can be so hard on your throad, it’s very difficult to inhale.  So additives are used to reduce the smoke’s harshness.  But even though you can’t feel the true harshness of the smoke, the effect it’s having on your body is still harsh…  Barb talks about her experience with lung cancer here: xxx.tiny.url |

| All smoke is toxic | | |
| --- | --- | --- |
| **Burning the tobacco itself creates most of the toxic chemicals in cigarette smoke.** | Quitting smoking means yous top filling your body with toxic chemicals.  You CAN quit smoking. Call Quitline 13 7848 | **That is why all tobacco smoke is toxic for your body.**  It doesn’t matter if the cigarettes you smoke are:   - Tailor made or roll-your own - Menthol or non-menthol - Smooth or harsh - Light or strong - Cheap or expensive - “Organic” or not   The best way to protect yourself is to quit. |
| Light smoke, heavy on tar | | |
| **Cigarette smoke that feels light can still be heavy on tar.** | When you inhale, particles in the smoke condense to form a sticky layer of tar on the inside of your lungs. | **Tar damages your DNA.**  Smoke from some cigarettes feels lighter because tiny holes around the filter mix air with the smoke.  Even though this smoke feels lighter, you can end up inhaling as much tar as from stronger cigarettes.  When tar damages your DNA, it can cause lung cancer.  For help quitting, call Quitline 13 7848 |
| Light smoke, cancerous chemicals | | |
| **Cigarette smoke that feels light still contains cancer causing chemicals.** | Chemicals in the smoke change your DNA, causing mutations in your genes. These mutations can be the start of cancer.  For help quitting, call Quitline 13 7848 | Smoke from some cigarettes feels lighter because tiny holes around the filter mix air with the smoke.  This diluted smoke contains less nicotine.  So to get the nicotine hit they need, smokers of these cigarettes tend to inhale a larger volume of smoke by:   - covering up the holes - smoking harder or more often - smoking more cigarettes   No matter how the smoke feels, all smokers inhale chemicals that cause cancer. |

| Menthol makes smoking addictive | | |
| --- | --- | --- |
| **Menthol makes smoking more addictive.** | Menthol increases the number of nicotine receptors in your brain. This makes your brain more sensitive to nicotine. | **Fresh-tasting menthol helps nicotine get a hold on you.**  Menthol may make tobacco taste fresh.  But it also increases the effects of nicotine in your brain.  It makes you more addicted and that can make it harder to quit.  You CAN quit smoking.  For help quitting, call Quitline 13 7848 |
| Menthol addicts new smokers | | |
| **Menthol helps to addict new smokers.** | Menthol makes nicotine stay in your body for longer, increasing the time it can affect the brain. This may increase the ”reward” effects of nicotine on the brain. | **Menthol’s fresh taste helps the smoke go down.**  The fresh taste of menthol hides the harshness of tobacco smoke.  Menthol also increases the effect of nicotine in the brain.  Young people find the smoke easier to inhale and they become addicted to smoking more easily.  You CAN quit smoking.  For help quitting, call Quitline 13 7848 |
| Menthol numbs the throat | | |
| **Menthol numbs your throat so you can inhale poisons.** | Menthol dulls pain receptors in your mouth and throat. This can hide the pain and prevent the natural cough reflex normally triggered by harsh tobacco smoke. | Menthol cigarettes can feel soothing to smoke because menthol numbs your throat.  When your throat is numb, your body cannot tell how damaging the harsh smoke really is.  So you keep inhaling the toxic poisons that cause throat cancer.  Scott’s experience of throat cancer is here xxx.tiny.url  For help quitting, call Quitline 13 7848 |
| RYO factory processing | | |
| **Far from being natural, roll-your-own tobacco is processed in a factory.** | Roll-your-own tobacco looks and feels different to tailor-made cigarette tobacco. That is because it is cut into longer and thinner strips, and more additives are used to keep it moist.  For help quitting, call Quitline 13 7848 | **During factory processing, your tobacco can be mixed with slurry and puffed tobacco pieces.**  Slurry: a liquid made from ground-up tobacco stems and scraps mixed with water, binding agents and additives. The slurry is then dried into a sheet and shredded to look like tobacco.  Puffed tobacco: pieces of tobacco that are puffed up with gas to make them bigger.  Roll-your-own and tailor-made cigarette tobacco are both highly processed products. |
| RYO additives | | |
| **Over 10% of the weight of roll-your-own tobacco is from additives.** | Roll-your-own tobacco contains at least as many additives by weight as the tobacco in tailor-made cigarettes. | **Roll-your-own tobacco may seem natural. But additives are used to:**   - keep the tobacco moist - change the taste of the smoke - hide the smoke’s natural harshness   However, these additives can’t hide the harsh reality of the damage caused by smoking…such as mouth cancer.  You CAN quit smoking. Call Quitline 13 7848 |
| Burning moist RYO creates acrolein | | |
| **Burning moist tobacco creates toxic acrolein gas.** | Acrolein causes inflammation and other damage to your heart muscle. It increases your blood pressure and heart rate and can lead to heart failure. | **Chemicals called humectants are added to roll-your-own tobacco to keep it moist.**  When humectants burn, some of them produce a toxic gas called acrolein. Acrolein is also found in chemical weapons.  When you inhale the smoke, you inhale acrolein. Inhaling acrolein damages your heart.  Your doctor can advise you about medication that can help with quitting. |

Table S2. Product Attribute Health Warnings (HWs) potentially seen by participants in the Product Attribute HWs and Product Attribute HWs + Video conditions, by predominant product use.

|  | Predominantly FMC smokers | Predominantly RYO smokers | Menthol/Crushball smokers |
| --- | --- | --- | --- |
| PAHW 1 | Combustion creates new toxins | Combustion creates new toxins | Combustion creates new toxins |
| PAHW 2 | Additives hide true harshness | Additives hide true harshness | Additives hide true harshness |
| PAHW 3 | Light smoke, cancerous chemicals | RYO factory processing | Menthol makes smoking addictive |
| PAHW 4 | *One of:*  RYO factory processing;  RYO additives;  Burning moist RYO creates acrolein | RYO additives | Menthol addicts new smokers |
| PAHW 5 | *One of:*  Menthol makes smoking addictive;  Menthol addicts new smokers;  Menthol numbs the throat | *One of:*  Light smoke, cancerous chemicals; Light smoke, heavy on tar | *If FMC smoker, one of:*  Light smoke, cancerous chemicals; Light smoke, heavy on tar |
|  |  |  | *If RYO smoker, one of:* RYO factory processing;  RYO additives;  Burning moist RYO creates acrolein |
| PAHW 6 | Light smoke, heavy on tar | Burning moist RYO creates acrolein | Menthol numbs the throat |
| PAHW 7 | All smoke is toxic | All smoke is toxic | All smoke is toxic |

Note: PAHW, Product Attribute Health Warning; FMC, factory made cigarettes; RYO, roll-your-own cigarettes.

Table S3. Full description and transcript of the 30-second and 15-second videos shown during the baseline survey and repeated exposure tasks as part of the Product Attribute HWs+ Video condition.

|  | Transcript | Description |
| --- | --- | --- |
| 30-second video | “Inhaling the smoke from burning raw tobacco would feel like this [referring to imagery of rusted barbed wire], so tobacco companies con you by using masking agents, flavours, and modified filters to hide the harsh feel and taste.”  “But just because you can’t feel it, doesn’t mean the burning tobacco isn’t damaging you.”  “Tobacco companies know just how harsh and dangerous their products are, they just don’t want you to feel it. [Coughing sound]”  “It’s the con that kills.” | The video opens with close-up visuals of rusted barbed wire with a dark, black background. A rectangle of cotton wool is slowly wrapped around the barbed wire, before a filter and cigarette paper is added so the barbed wire is concealed within a cigarette. A man appears to then inhale from the cigarette, and the rusted barbed wire is seen to be drawn into the man’s mouth as it burns. The final screen shows text that states “IT’S THE CON THAT KILLS” on a black background, along with the cigarette containing rusted barbed wire. |
| 15-second video, version A | “Inhaling the smoke from burning raw tobacco would feel like this [referring to imagery of rusted barbed wire], so tobacco companies con you by using masking agents to hide the harsh feel and taste.”  “It’s the con that kills.” | As described above for the 30-second video, excluding the imagery of the man inhaling from the cigarette. |
| 15-second video, version B | “Inhaling the smoke from burning raw tobacco would feel like this [referring to imagery of rusted barbed wire], so tobacco companies con you by using modified filters to hide the harsh feel and taste.”  “It’s the con that kills.” | As described above for the 30-second video, excluding the imagery of the man inhaling from the cigarette. |

Table S4. Number and proportion of participants with each number of repeated exposure tasks (RETs) completed among participants who completed the follow-up survey, by condition.

|  | Control | Standard HWs | Product Attribute HWs | Product Attribute HWs + Video | |
| --- | --- | --- | --- | --- | --- |
|  | Proportion who completed each number of RETs | Proportion who completed each number of RETs | Proportion who completed each number of RETs | Proportion who completed each number of RETs | Proportion with each number of video exposures during RETs |
|  | n (%) | n (%) | n (%) | n (%) | n (%) |
| 0 | 32 (8.8) | 27 (7.9) | 22 (6.2) | 30 (8.4) | 34 (9.6) |
| 1 | 40 (11.1) | 19 (5.6) | 28 (7.9) | 39 (11.0) | 42 (11.8) |
| 2 | 28 (7.7) | 37 (10.9) | 36 (10.1) | 29 (8.2) | 31 (8.7) |
| 3 | 50 (13.8) | 36 (10.6) | 54 (15.2) | 42 (11.8) | 49 (13.8) |
| 4 | 61 (16.9) | 63 (18.5) | 67 (18.8) | 66 (18.5) | 71 (19.9) |
| 5 | 66 (18.2) | 68 (20.0) | 59 (16.6) | 61 (17.1) | 67 (18.8) |
| 6 | 47 (13.0) | 49 (14.4) | 46 (12.9) | 41 (11.5) | 62 (17.4) |
| 7 | 38 (10.5) | 41 (12.1) | 44 (12.4) | 48 (13.5) | - |

Note: The health warnings (HWs) were shown at all repeated exposure tasks (RETs). For Product Attribute HWs + Video participants, the video was also shown during all RETs except the first RET, hence these participants could be exposed to the video a maximum of six times. Proportions are rounded so may not sum to 100.0%. Base: Analytic sample of participants who completed the follow-up survey.

Table S5. Results from a multivariable regression model to predict participation in the follow-up survey.

|  | Study sample  N=2,347 | |
| --- | --- | --- |
|  | PR (95%CI) | *p*-value |
| *Condition* |  |  |
| Control | 1.00 (ref) | - |
| Standard HWs | 0.95 (0.87, 1.04) | 0.245 |
| Product Attribute HWs | 0.97 (0.89, 1.05) | 0.449 |
| Product Attribute HWs + Video | 0.98 (0.90, 1.06) | 0.584 |
| *Number of RETs completed* | **1.24 (1.22, 1.26)** | **<0.001***** |
| *Age group* |  |  |
| 18-39 years | 1.00 (ref) | - |
| 40-54 years | 1.07 (1.00, 1.13) | **0.037*** |
| 55-69 years | 0.96 (0.88, 1.06) | 0.425 |
| *Gender* |  |  |
| Man or male | 1.00 (ref) | - |
| Woman or female | **1.14 (1.08, 1.21)** | **<0.001***** |
| *Highest level of education* |  |  |
| No tertiary education | 1.00 (ref) | - |
| Tertiary education | 0.95 (0.89, 1.01) | 0.078 |
| *Socio-economic area* |  |  |
| Low | 1.00 (ref) | - |
| Mid | 1.01 (0.96, 1.07) | 0.666 |
| High | 0.92 (0.85, 1.01) | 0.075 |
| *Geographic region* |  |  |
| Metropolitan | 1.00 (ref) | - |
| Regional | 0.99 (0.93, 1.05) | 0.639 |
| *Aboriginal and/or Torres Strait Islander* |  |  |
| No | 1.00 (ref) | - |
| Yes | 0.92 (0.80, 1.05) | 0.222 |
| *Health care card or pensioner concession card holder ^b^* |  |  |
| No | 1.00 (ref) | - |
| Yes | 1.05 (0.99, 1.12) | 0.076 |
| *Quit attempts in past year ^c^* |  |  |
| None | 1.00 (ref) | - |
| At least once | **1.06 (1.00, 1.13)** | **0.039*** |
| Don’t know / can’t say | 1.10 (0.98, 1.23) | 0.101 |
| *Frequency of e-cigarette use* |  |  |
| Less than monthly | 1.00 (ref) | - |
| At least monthly | 1.00 (0.94, 1.06) | 0.983 |
| *Frequency of FMC and/or RYO cigarette use* |  |  |
| Less than daily | 1.00 (ref) | - |
| At least daily | 1.03 (0.96, 1.10) | 0.480 |
| *Predominant product use ^d^* |  |  |
| RYO | 1.00 (ref) | - |
| FMC | 0.96 (0.90, 1.02) | 0.221 |
| Menthol/menthol crushball RYO | 0.99 (0.89, 1.11) | 0.913 |
| Menthol/menthol crushball FMC | 0.92 (0.83, 1.01) | 0.088 |
| *Knowledge of harms despite sensory experiences* | 1.00 (0.95, 1.06) | 0.892 |
| *Knowledge of tobacco industry manipulation* | 0.98 (0.93, 1.03) | 0.420 |
| *Self-centric negative emotional responses* | 0.99 (0.95, 1.03) | 0.485 |
| *Industry-centric negative emotional responses* | 1.01 (0.98, 1.05) | 0.394 |

Note: To maximise sample size in the regression model, missing or non-binary data for gender (n=10) was back-coded into each of the two categories listed above, missing data for highest level of education (n=17) was back-coded into the most common category listed above (no tertiary education), and missing data for socio-economic area (n=1) was back-coded into the most common category listed above (mid).

Abbreviations: HWs=health warnings; RETs=repeated exposure tasks; FMC=factory-made cigarettes; RYO=roll-your-own

Table S6. Condition assignment, socio-demographic and smoking characteristics of the study sample measured pre-exposure at baseline (N=2,544) and follow-up (N=1,414) compared with Australian population data from the 2019 National Drug Strategy Household Survey.

|  | Baseline sample | Follow-up sample | Australian population |
| --- | --- | --- | --- |
|  | n (%) | n (%) | n (%) |
| *Condition* |  |  |  |
| Control | 637 (25.0) | 362 (25.6) | - |
| Standard HWs | 637 (25.0) | 340 (24.0) | - |
| Product Attribute HWs | 636 (25.0) | 356 (25.2) | - |
| Product Attribute HWs + Video | 634 (24.9) | 356 (25.2) | - |
| *Age group* |  |  |  |
| 18-39 years | 1,516 (59.6) | 790 (55.9) | 1,298 (45.1) |
| 40-54 years | 742 (29.2) | 453 (32.0) | 982 (34.1) |
| 55-69 years | 286 (11.2) | 171 (12.1) | 599 (20.8) |
| *Gender ^a^* |  |  |  |
| Man / male | 1,265 (49.7) | 610 (43.1) | 1,604 (55.7) |
| Woman / female | 1,269 (49.9) | 799 (56.5) | 1,274 (44.3) |
| Another term | 5 (0.2) | 3 (0.2) | - |
| Prefer not to say | 5 (0.2) | 2 (0.1) | - |
| *Highest level of education* |  |  |  |
| No tertiary education | 1,441 (56.6) | 861 (60.9) | 1,975 (74.5) |
| Tertiary education | 1,086 (42.7) | 543 (38.4) | 675 (25.5) |
| *Socio-economic area* |  |  |  |
| Low | 987 (38.8) | 580 (41.0) | 1,495 (51.9) |
| Mid | 1,043 (41.0) | 598 (42.3) | 1,037 (36.0) |
| High | 513 (20.2) | 236 (16.7) | 347 (12.0) |
| *Geographic region* |  |  |  |
| Metropolitan | 1,840 (72.3) | 993 (70.2) | 1,878 (65.2) |
| Regional | 704 (27.7) | 421 (29.8) | 1,001 (34.8) |
| *Aboriginal and/or Torres Strait Islander* |  |  |  |
| No | 2,365 (93.0) | 1,338 (94.6) | 2,708 (94.1) |
| Yes | 150 (5.9) | 68 (4.8) | 156 (5.4) |
| Prefer not to say | 29 (1.1) | 8 (0.6) | 15 (0.5) |
| *Health care card or pensioner concession card holder ^b^* |  |  |  |
| No | 1,681 (66.1) | 901 (63.7) | - |
| Yes | 863 (33.9) | 513 (36.3) | - |
| *Quit attempts in past year ^c^* |  |  |  |
| None | 1,178 (46.3) | 620 (43.9) | 1,331 (46.2) |
| At least once | 1,179 (46.3) | 691 (48.9) | 1,502 (52.2) |
| Don’t know / can’t say | 187 (7.4) | 103 (7.3) | 46 (1.6) |
| *Frequency of e-cigarette use* |  |  |  |
| Less than monthly | 1,490 (58.6) | 881 (62.3) | 2,620 (92.0) |
| At least monthly | 1,054 (41.4) | 533 (37.7) | 229 (8.0) |
| *Frequency of FMC and/or RYO cigarette use* |  |  |  |
| Less than daily | 568 (22.3) | 292 (20.7) | 615 (21.4) |
| At least daily | 1,976 (77.7) | 1,122 (79.4) | 2,264 (78.6) |
| *Predominant product use ^d^* |  |  |  |
| RYO | 525 (20.6) | 322 (22.8) | 422 (22.0) |
| FMC | 1,411 (55.5) | 782 (55.3) | 1257 (65.6) |
| Menthol/menthol crushball RYO | 309 (12.2) | 152 (10.8) | 42 (2.2) |
| Menthol/menthol crushball FMC | 299 (11.8) | 158 (11.2) | 194 (10.1) |

Notes: Proportions are rounded so may not sum to 100.0%. No response was provided for highest level of education for n=17 among the baseline sample and n=10 among the 8-day follow-up sample. Socio-economic area could not be computed for n=1 among the baseline sample. Abbreviations: HWs=health warnings; FMC=factory-made cigarettes; RYO=roll-your-own

^a^ The study sample for the 2019 National Drug Strategy Household Survey excludes n=115 respondents who used another term to describe their gender.

^b^ Individual-level measure of socio-economic status. Data not available as part of the 2019 National Drug Strategy Household Survey.

^c^ Participants in the 2019 National Drug Strategy Household Survey who had made at least one quit attempt in the past year were defined as those who made at least one unsuccessful quit attempt or those who made a successful quit attempt but were current smokers at the time of the interview (i.e., had relapsed).

^d^ Comparative data were sourced from the 2022 Victorian Smoking and Health Survey. Respondents who smoked FMC or RYO were categorised as to whether they ‘always’ smoked menthol cigarettes or tobacco. However, the survey did not specifically ask whether respondents smoked cigarettes with crushable menthol capsules in the filter, so would underestimate the prevalence of overall menthol use.

Table S7. Descriptive statistics for primary outcomes at follow-up by condition and effect sizes for comparisons between the control and intervention conditions among participants who predominantly smoked tailor-made cigarettes.

|  | Control ^†^  n=208 | Standard HWs condition  n=183 | | Product Attribute HWs condition  n=191 | | Product Attribute HWs + Video  n=200 | | | Standard HWs cf. Product Attribute HWs post-estimation comparison | Product Attribute HWs cf. Product Attribute HWs + Video post-estimation comparison |
| --- | --- | --- | --- | --- | --- | --- | --- | --- | --- | --- |
|  | Mean change | Mean change | Time $\times$ condition interaction β (95%CI) | Mean change | Time $\times$ condition interaction β (95%CI) | Mean change | Time $\times$ condition interaction β (95%CI) | | *p* | *p* |
| *Favourable sensory cues* |  |  |  |  |  |  |  | |  |  |
| Enjoyment | -0.58 | -0.99 | -0.34 (-3.50, 2.82) | -0.51 | -0.08 (-3.21, 3.04) | -4.64 | **-4.06 (-7.16, -0.96)*** | | - | **0.013*** |
| Concern | 3.55 | 4.37 | 2.14 (-2.55, 6.83) | 8.49 | **6.06 (1.43, 10.70)*** | 4.95 | 2.96 (-1.63, 7.56) | | 0.106 | 0.193 |
| *Unfavourable sensory cues* |  |  |  |  |  |  |  | |  |  |
| Enjoyment | -0.82 | -0.35 | 0.36 (-2.42, 3.15) | -2.36 | -1.61 (-4.36, 1.14) | -1.76 | -0.05 (-2.78, 2.68) | | - | - |
| Concern | 1.41 | 3.90 | 3.00 (-0.90 (6.91) | 3.20 | **3.98 (0.13, 7.84)*** | 2.23 | 1.15 (-2.68, 4.98) | | 0.628 | 0.153 |
|  |  |  |  |  |  |  | |  |  |  |
|  | Mean | Mean | β (95%CI) | Mean | β (95%CI) | Mean | | β (95%CI) | *p* | *p* |
| *Knowledge* |  |  |  |  |  |  | |  |  |  |
| Knowledge of harms despite sensory experiences | 4.16 | 4.19 | 0.03 (-0.11, 0.17) | 4.38 | **0.22 (0.08, 0.35)**** | 4.22 | | 0.06 (-0.07, 0.20) | **0.010*** | **0.032*** |
| Knowledge of tobacco industry manipulation | 3.98 | 3.95 | -0.04 (-0.18, 0.11) | 4.19 | **0.21 (0.07, 0.35)*** | 4.12 | | 0.14 (0.00, 0.28)^ | **0.001**** | 0.325 |
| *Negative emotional responses* |  |  |  |  |  |  | |  |  |  |
| Self-centric negative emotional responses | 3.02 | 3.44 | **0.42 (0.25, 0.59)***** | 3.53 | **0.50 (0.33, 0.67)***** | 3.46 | | **0.44 (0.27, 0.61)***** | 0.339 | 0.462 |
| Industry-centric negative emotional responses | 2.89 | 3.12 | **0.23 (0.03, 0.42)*** | 3.37 | **0.47 (0.29, 0.66)***** | 3.43 | | **0.54 (0.35, 0.72)***** | **0.013*** | 0.531 |
| *Product-specific smoking dissonance* | 3.21 | 3.31 | 0.10 (-0.07, 0.27) | 3.43 | **0.22 (0.06, 0.38)*** | 3.29 | | 0.08 (-0.08, 0.24) | 0.166 | 0.094 |
|  | % | % | PR (95% CI) | % | PR (95% CI) | % | | PR (95% CI) | *p* | *p* |
| *Continued engagement with HWs* |  |  |  |  |  |  | |  |  |  |
| Past-week rumination about HWs | 13.9 | 25.7 | **1.84 (1.21, 2.80)**** | 33.0 | **2.37 (1.60, 3.51)***** | 27.5 | | **1.97 (1.31, 2.96)**** | 0.124 | 0.239 |
| Past-week online HW information-seeking | 6.3 | 10.4 | 1.66 (0.84, 3.27) | 13.5 | **2.18 (1.15, 4.12)*** | 14.5 | | **2.32 (1.24, 4.33)*** | 0.340 | 0.801 |
| Past-week interpersonal discussion about HWs | 12.5 | 16.4 | 1.31 (0.81, 2.13) | 23.0 | **1.84 (1.18, 2.87)*** | 25.5 | | **2.04 (1.33, 3.14)**** | 0.110 | 0.571 |
| *Smoke-limiting micro behaviours* |  |  |  |  |  |  | |  |  |  |
| Tried to limit the number of cigarettes smoked in past week | 61.5 | 71.6 | 1.16 (1.01, 1.34)^ | 68.1 | 1.11 (0.96, 1.28) | 65.0 | | 1.06 (0.91, 1.22) | - | - |
| Stubbed/butted out cigarette before finishing it in past week | 60.1 | 64.5 | 1.07 (0.92, 1.25) | 63.9 | 1.06 (0.91, 1.24) | 65.0 | | 1.08 (0.93, 1.26) | - | - |
| Forgone a cigarette in past week | 48.6 | 54.6 | 1.13 (0.93, 1.36) | 55.0 | 1.13 (0.94, 1.37) | 59.0 | | 1.22 (1.01, 1.46)^ | - | - |
| At least one of the above smoke-limiting micro behaviours | 79.3 | 86.3 | 1.09 (0.99, 1.19) | 84.3 | 1.06 (0.97, 1.17) | 85.0 | | 1.07 (0.98, 1.17) | - | - |

Note: Statistically significant difference at ****p*<0.001, ***p*<0.010, and **p*<0.05. All *p*-values have been adjusted for multiple comparisons using the Holm-Bonferroni method, with the exception of post-estimation comparison *p*-values. Where significant *p*-values became non-significant after adjustment, this has been indicated (^). Maximum sample sizes are reported; sample sizes differ slightly by measure. All regression models are unadjusted. † Reference category. Abbreviations: HWs=health warnings; PR = prevalence ratio.

Table S8. Descriptive statistics for primary outcomes at follow-up by condition and effect sizes for comparisons between the control and intervention conditions among participants who had not completed any tertiary education.

|  | Control ^†^  n=221 | Standard HWs condition  n=212 | | Product Attribute HWs condition  n=208 | | Product Attribute HWs + Video  n=220 | | | Standard HWs cf. Product Attribute HWs post-estimation comparison | Product Attribute HWs cf. Product Attribute Health Warnings + Video post-estimation comparison |
| --- | --- | --- | --- | --- | --- | --- | --- | --- | --- | --- |
|  | Mean change | Mean change | Time $\times$ condition interaction β (95%CI) | Mean change | Time $\times$ condition interaction β (95%CI) | Mean change | Time $\times$ condition interaction β (95%CI) | | *p* | *p* |
| *Favourable sensory cues* |  |  |  |  |  |  |  | |  |  |
| Enjoyment | -1.26 | -0.76 | 0.12 (-2.80, 3.04) | -2.17 | -1.05 (-3.99, 1.89) | -3.28 | -2.97 (-5.87, -0.07)^ | | - | - |
| Concern | 4.60 | 4.51 | 0.58 (-3.86, 5.01) | 7.96 | 4.79 (0.34, 9.24)^ | 8.52 | **5.53 (1.13, 9.93)*** | | - | 0.745 |
| *Unfavourable sensory cues* |  |  |  |  |  |  |  | |  |  |
| Enjoyment | -1.33 | -1.97 | -0.88 (-3.39, 1.64) | -2.14 | -0.79 (-3.32, 1.74) | -3.27 | -1.64 (-4.14, 0.85) | | - | - |
| Concern | 1.45 | 3.79 | 2.62 (-1.26, 6.50) | 2.86 | 2.94 (-0.96, 6.83) | 0.65 | -0.09 (-3.95, 3.76) | | - | - |
|  |  |  |  |  |  |  | |  |  |  |
|  | Mean | Mean | β (95%CI) | Mean | β (95%CI) | Mean | | β (95%CI) | *p* | *p* |
| *Knowledge* |  |  |  |  |  |  | |  |  |  |
| Knowledge of harms despite sensory experiences | 4.25 | 4.25 | 0.01 (-0.13, 0.14) | 4.32 | 0.07 (-0.06, 0.21) | 4.26 | | 0.01 (-0.12, 0.14) | - | - |
| Knowledge of tobacco industry manipulation | 4.02 | 4.00 | -0.02 (-0.16, 0.11) | 4.12 | 0.10 (-0.03, 0.23) | 4.13 | | 0.11 (-0.02, 0.24) | - | - |
| *Negative emotional responses* |  |  |  |  |  |  | |  |  |  |
| Self-centric negative emotional responses | 3.05 | 3.43 | **0.38 (0.21, 0.55)***** | 3.42 | **0.37 (0.20, 0.54)***** | 3.42 | | **0.37 (0.20, 0.54)***** | 0.914 | 1.00 |
| Industry-centric negative emotional responses | 2.95 | 3.16 | **0.22 (0.03, 0.40)*** | 3.31 | **0.36 (0.18, 0.55)***** | 3.34 | | **0.40 (0.22, 0.58)***** | 0.118 | 0.723 |
| *Product-specific smoking dissonance* | 3.24 | 3.30 | 0.06 (-0.10, 0.22) | 3.32 | 0.07 (-0.09, 0.23) | 3.25 | | 0.01 (-0.15, 0.17) | - | - |
|  | % | % | PR (95% CI) | % | PR (95% CI) | % | | PR (95% CI) | *p* | *p* |
| *Continued engagement with HWs* |  |  |  |  |  |  | |  |  |  |
| Past-week rumination about HWs | 15.8 | 22.2 | 1.40 (0.94, 2.08) | 26.4 | **1.67 (1.14, 2.44)*** | 31.4 | | **1.98 (1.38, 2.84)***** | 0.309 | 0.264 |
| Past-week online HW information-seeking | 7.2 | 11.8 | 1.63 (0.89, 2.96) | 8.7 | 1.20 (0.63, 2.28) | 13.2 | | 1.82 (1.02, 3.26)^ | - | - |
| Past-week interpersonal discussion about HWs | 15.4 | 21.2 | 1.38 (0.92, 2.07) | 17.3 | 1.13 (0.73, 1.73) | 25.0 | | **1.63 (1.11, 2.39)*** | - | 0.055 |
| *Smoke-limiting micro behaviours* |  |  |  |  |  |  | |  |  |  |
| Tried to limit the number of cigarettes smoked in past week | 66.5 | 71.2 | 1.07 (0.94, 1.22) | 67.8 | 1.02 (0.89, 1.16) | 65.0 | | 0.98 (0.85, 1.12) | - | - |
| Stubbed/butted out cigarette before finishing it in past week | 62.4 | 63.7 | 1.02 (0.88, 1.18) | 59.6 | 0.95 (0.82, 1.11) | 68.6 | | 1.10 (0.96, 1.26) | - | - |
| Forgone a cigarette in past week | 55.7 | 55.2 | 0.99 (0.84, 1.17) | 56.7 | 1.02 (0.86, 1.20) | 58.2 | | 1.05 (0.89, 1.23) | - | - |
| At least one of the above smoke-limiting micro behaviours | 85.1 | 87.3 | 1.03 (0.95, 1.11) | 84.1 | 0.99 (0.91, 1.07) | 85.5 | | 1.00 (0.93, 1.09) | - | - |

Note: Statistically significant difference at ****p*<0.001, ***p*<0.010, and **p*<0.05. All *p*-values have been adjusted for multiple comparisons using the Holm-Bonferroni method, with the exception of post-estimation comparison *p*-values. Where significant *p*-values became non-significant after adjustment, this has been indicated (^). Maximum sample sizes are reported; sample sizes differ slightly by measure. All regression models are unadjusted. † Reference category. Abbreviations: HWs=health warnings; PR = prevalence ratio.

Table S9. Descriptive statistics for primary outcomes immediately post-exposure by condition and effect sizes for comparisons between the control and intervention conditions.

|  | Control ^†^  n=637 | Standard HWs condition  n=637 | | Product Attribute HWs condition  n=636 | | Product Attribute HWs + Video  n=634 | | Standard HWs cf. Product Attribute HWs post-estimation comparison | Product Attribute HWs cf. Product Attribute HWs + Video post-estimation comparison |
| --- | --- | --- | --- | --- | --- | --- | --- | --- | --- |
|  | Mean | Mean | β (95%CI) | Mean | β (95%CI) | Mean | β (95%CI) | *p* | *p* |
| *Knowledge* |  |  |  |  |  |  |  |  |  |
| Knowledge of harms despite sensory experiences | 4.10 | 4.16 | 0.06 (-0.02, 0.13) | 4.18 | 0.08 (0.01, 0.16)^ | 4.20 | **0.10 (0.02, 0.18)*** | - | 0.675 |
| Knowledge of tobacco industry manipulation | 3.87 | 3.99 | **0.12 (0.04, 0.19)**** | 4.05 | **0.18 (0.10, 0.25)***** | 4.09 | **0.21 (0.14, 0.29)***** | 0.124 | 0.321 |
| *Negative emotional responses* |  |  |  |  |  |  |  |  |  |
| Self-centric negative emotional responses | 2.60 | 3.64 | **1.04 (0.95, 1.14)***** | 3.48 | **0.88 (0.79, 0.98)***** | 3.50 | **0.90 (0.81, 1.00)***** | **0.001**** | 0.721 |
| Industry-centric negative emotional responses | 2.68 | 3.17 | **0.49 (0.38, 0.59)***** | 3.27 | **0.59 (0.48, 0.70)***** | 3.43 | **0.75 (0.64, 0.86)***** | 0.055 | **0.005**** |

Note: Statistically significant difference at ****p*<0.001, ***p*<0.010, and **p*<0.05. All *p*-values have been adjusted for multiple comparisons using the Holm-Bonferroni method, with the exception of post-estimation comparison *p*-values. Where significant *p*-values became non-significant after adjustment, this has been indicated (^). All regression models are unadjusted. † Reference category. Abbreviations: HW=health warning.
